# Myosin-based nucleation of actin filaments contributes to stereocilia development critical for hearing

**DOI:** 10.1038/s41467-025-55898-8

**Published:** 2025-01-22

**Authors:** Zane G. Moreland, Fangfang Jiang, Carlos Aguilar, Melanie Barzik, Rui Gong, Ghazaleh Behnammanesh, Jinho Park, Arik Shams, Christian Faaborg-Andersen, Jesse C. Werth, Randall Harley, Daniel C. Sutton, James B. Heidings, Stacey M. Cole, Andrew Parker, Susan Morse, Elizabeth Wilson, Yasuharu Takagi, James R. Sellers, Steve D. M. Brown, Thomas B. Friedman, Gregory M. Alushin, Michael R. Bowl, Jonathan E. Bird

**Affiliations:** 1 Department of Pharmacology and Therapeutics, University of Florida, Gainesville, FL USA; 2 Myology Institute, University of Florida, Gainesville, FL USA; 3 Graduate Program in Biomedical Sciences, University of Florida, Gainesville, FL USA; 4 McKnight Brain Institute, University of Florida, Gainesville, FL USA; 5 Mammalian Genetics Unit, MRC Harwell Institute, Didcot, UK; 6 Laboratory of Molecular Genetics, National Institute on Deafness and Other Communication Disorders, National Institutes of Health, Bethesda, MD USA; 7 Laboratory of Structural Biophysics and Mechanobiology, The Rockefeller University, New York, NY USA; 8 Laboratory of Molecular Physiology, Cell and Developmental Biology Center, National Heart, Lung and Blood Institute, National Institutes of Health, Bethesda, MD USA; 9 UCL Ear Institute, University College London, London, UK; 10 Present Address: UCL Ear Institute, University College London, London, UK

**Keywords:** Myosin, Hair cell, Enzyme mechanisms

## Abstract

Assembly of actin-based stereocilia is critical for cochlear hair cells to detect sound. To tune their mechanosensivity, stereocilia form bundles composed of graded rows of ascending height, necessitating the precise control of actin polymerization. Myosin 15 (MYO15A) drives hair bundle development by delivering critical proteins to growing stereocilia that regulate actin polymerization via an unknown mechanism. Here, we show that MYO15A is itself an actin nucleation-promoting factor. Moreover, a deafness-causing mutation in the MYO15A actin-binding interface inhibits nucleation activity but still preserves some movement on filaments in vitro and partial trafficking on stereocilia in vivo. Stereocilia fail to elongate correctly in this mutant mouse, providing evidence that MYO15A-driven actin nucleation contributes to hair bundle biogenesis. Our work shows that in addition to generating force and motility, the ATPase domain of MYO15A can directly regulate actin polymerization and that disrupting this activity can promote cytoskeletal disease, such as hearing loss.

## Abstract (web summary)

Actin filament polymerization is crucial for building sound-sensitive stereocilia in the cochlea. Here, the authors show that a myosin motor can nucleate actin filaments, revealing a mechanism for stereocilia growth and hereditary hearing loss.

## Introduction

Cochlear hair cells are the primary transducers of sound in the mammalian inner ear and are fundamental for hearing. Each hair cell assembles approximately 100 individual stereocilia on its apical surface to form a mechano-sensitive hair bundle. Within each bundle, stereocilia are precisely graded into ranks of ascending height and this staircase architecture is critical for mechano-electric transduction (MET)^1^. The regulation of stereocilia size is thus central for hearing and the disruption of hair bundle architecture is a common theme in hereditary deafness^2^. Stereocilia develop from microvilli by building a para-crystalline core of highly cross-linked actin filaments as an internal scaffold to confer shape and structural rigidity^3^. As stereocilia develop, the actin core thickens and elongates to reach its mature size, necessitating precise control of actin filament polymerization^4–6^. Actin filaments are uniformly polarized within stereocilia with rapidly growing barbed ends orientated towards the tip, the major site of actin polymerization and filament elongation, and also the site of MET^7–9^. Once stereocilia are fully assembled, actin polymerization continues primarily at the tip compartment, indicating an ongoing plasticity throughout adult life^10–13^. A number of proteins have been identified within the tip compartment that are essential for stereocilia growth, yet the molecular mechanisms governing actin polymerization are unknown. Identification of this mechanism is central to the acquisition and maintenance of hair cell mechano-sensitivity, and also for understanding hearing loss as the clinical manifestation of a cytoskeletal disease.

**Fig. 1:**
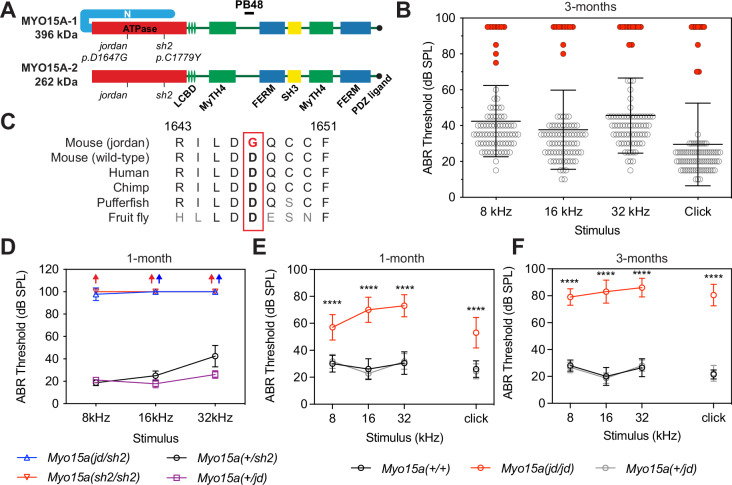
The *jordan* mutation causes progressive hearing loss in a mouse model of DFNB3. **A** Schematic showing the protein domains of the long (MYO15A-1) and short (MYO15A-2) isoforms encoded by the *Myo15a* gene. The *jordan* and *shaker-2* deafness mutations are shown. **B** ABR phenotyping of the *jordan* pedigree at 3 months identified 10 mice with statistically-elevated hearing thresholds (red circles) for click and at 8, 16, and 32 kHz stimuli, compared to their normal hearing pedigree mates (*n* = 73 mice, black circles). Statistical outliers were detected using robust regression and outlier removal (red circles, ROUT, *Q* = 1%). Thresholds of affected mice that did not respond to the highest intensity stimulus (90 dB SPL) are recorded as 95 dB SPL. **C** Evolutionary conservation of the aspartate (D) residue of MYO15A altered to glycine (G) in *jordan* mice that causes hearing loss. Residue positions refer to mouse MYO15A-1 (NP_034992.2). **D** ABR recordings of *Myo15a*^*jd/sh2*^ (*n* = 7 mice) compound heterozygotes at P28 shows profound hearing loss, similar to *Myo15a*^*sh2/sh2*^ (*n* = 4), with thresholds elevated compared with normal hearing *Myo15a*^*+/jd*^ (*n* = 9) *or Myo15a*^*+/sh2*^ littermates (*n* = 4). Arrows indicate no response. **E**, **F** Longitudinal auditory phenotyping of *jordan* mice at 1- (**E**) and 3- (**F**) months of age. ABR recordings show that *Myo15a* ^*jd/jd*^ mice (red, *n* = 10 mice) exhibit a progressive, moderate-to-severe hearing loss affecting all frequencies, whereas age-matched *Myo15a*^*+/+*^ (black, *n* = 10) and *Myo15a*^*+/jd*^ (grey, *n* = 15) littermate controls have normal thresholds (15–45 dB SPL). *Myo15a*^*+/+*^ *vs Myo15a*^*jd/jd*^ comparison, ****, *P* < 0.0001, ANOVA with Tukey’s multiple comparisons test. Data are mean ± SD.

A key molecule in establishing stereocilia architecture is unconventional myosin 15 (MYO15A), encoded by the gene *Myo15a* in mice and *MYO15A* in humans. Mutations in *MYO15A* (OMIM #602666) cause DFNB3 recessive hereditary hearing loss in humans^14–16^. MYO15A is a member of the myosin superfamily of P-loop ATPases that generate contractile force on actin filaments to power cellular processes such as cytokinesis, endocytosis and vesicular trafficking^17,18^. In addition to MYO15A, multiple other myosin proteins are required for normal stereocilia function and assembly, including MYO1C, MYO3A, MYO6, and MYO7A^19,20^. Two MYO15A isoforms are produced in auditory hair cells through alternative mRNA splicing^21–23^. A shorter isoform (MYO15A-2, also known as MYO15A-S) consists of the ATPase ‘motor domain’ and three light-chain binding sites that associate with calmodulin-like proteins, in addition to myosin tail homology 4 (MyTH4), Src homology 3 (SH3), and protein 4.1, ezrin, radixin, moesin (FERM) domains (Fig. 1A). A larger isoform (MYO15A-1, also known as MYO15A-L) is identical to MYO15A-2, except for the addition of a 133 kDa N-terminal domain (Fig. 1A). Both MYO15A isoforms localize to the stereocilia tip compartment where actin polymerization is concentrated^10,12,13,23,24^. The prototypical *Myo15a* mutant allele, the *shaker-2* (*Myo15a*^*sh2*^), prevents both isoforms from accumulating in stereocilia, blocking developmental elongation and causing profound hearing loss from birth^21,23–25^. The use of an isoform-specific *Myo15a-*null allele revealed additional functions, with MYO15A-2 being necessary for stereocilia developmental elongation, whilst MYO15A-1 independently maintains the postnatal size of shorter stereocilia^23^ that are structurally plastic in response to MET^6,26^.

The MYO15A-2 isoform associates with additional proteins essential for stereocilia elongation, and by inference, actin polymerization. The elongation complex (EC) consists of epidermal growth factor receptor pathway substrate 8 (EPS8), whirlin (WHRN), G-protein signalling modulator 2 (GPSM2) and G-protein subunit alpha_i3_ (GNAI3). Similar to MYO15A-2, these proteins are concentrated at the tips of the tallest stereocilia (row 1)^27–34^. Individual knock-out mouse mutants of *Eps8*, *Whrn*, *Gpsm2* or *Gnai3* phenocopy the *shaker-2* and exhibit short stereocilia along with profound deafness^27,30–34^. There is strong evidence that a key function of MYO15A-2 is to traffic the elongation complex and concentrate it at the stereocilia tips. First, elongation complex proteins are absent from the stereocilia of *Myo15a shaker-2* hair cells, demonstrating their functional dependence upon MYO15A-2 in vivo^28–30,32,34^. Second, EGFP-tagged MYO15A-2 actively traffics elongation complex proteins along filopodia in cell lines^28,30,32^. Finally, enzymatic studies of the purified MYO15A ATPase domain reveal kinetic adaptations that enable long-range processive molecular trafficking^35,36^. Together, these data support MYO15A-2 mediated delivery of the elongation complex to stereocilia tips, where the elongation complex is hypothesized to regulate actin polymerization. Despite its central role in promoting stereocilia growth, the specific molecular activity of the elongation complex remains unknown.

Here, we describe a *Myo15a* mutant mouse (‘*jordan*’) that exhibits progressive hearing loss resulting from a missense substitution in the MYO15A motor domain. In striking contrast with *shaker-2* hair cells, MYO15A and the elongation complex proteins are still detected at the stereocilia tip compartment in *jordan* mutant hair cells; however, in spite of this, their stereocilia fail to elongate properly. These results questioned the sufficiency of the elongation complex to drive stereocilia growth, and prompted us to search for another role of MYO15A, independent of delivering the elongation complex. We found that purified MYO15A motor domain protein directly stimulated actin polymerization in vitro, and that the *jordan* mutation blocked this activity, whilst only moderately affecting its ability to bind and move along actin filaments. A recently published study shows that the *jordan* mutation targets the actomyosin binding interface and interferes with the ability of wild-type MYO15A to regulate structural plasticity within the actin molecule itself ^37^. The structural insights from that work combined with our results below, suggest that in parallel with elongation complex activity, MYO15A has a critical role influencing stereocilia elongation by directly regulating F-actin conformation and stimulating actin polymerization at the stereocilia tip. More broadly, our work suggests that in addition to their classical roles generating force and motility, myosins have a physiological role regulating actin polymerization in vivo.

## Results

### A forward genetic screen identifies *jordan*, a *Myo15a* allele causing progressive hearing loss

During a phenotype-driven ENU-mutagenesis screen^38^, the MPC190 cohort (comprising 83 mice) was identified with 10 mice exhibiting severe hearing loss at 3 months of age (Fig. 1B). A genome scan and single nucleotide polymorphism (SNP) mapping of third generation (G3) deaf mice found linkage to a 16.7 Mb region on chromosome 11 (Supplementary Fig. 1A). Whole-genome sequencing of a single deaf mouse identified a high confidence homozygous mutation within the critical interval, consisting of an A-to-G transition at coding nucleotide 4940 of the *Myo*15a gene (ENSMUST00000071880). This variant was confirmed by Sanger sequencing (Supplementary Fig. 1B) and leads to the substitution of an evolutionarily conserved aspartate residue with a glycine (p.D1647G) in the encoded MYO15A protein (Fig. 1C). ClustalW alignments revealed that the residue D1647 of mouse MYO15A was broadly conserved, with acidic residues occupying the same position across other unconventional myosins, along with identity of some neighbouring residues (Supplementary Fig. 1C).

To confirm that the *Myo*15a^*jd*^ substitution causes hearing loss, we performed a genetic complementation test utilizing the *shaker-2* (*Myo15a*^*sh2*^) deafness allele in trans^25,39^. We recorded auditory brainstem responses (ABR) from postnatal day 28 (P28) mice and found that compound heterozygous *Myo15a*^*jd/sh2*^ mice had elevated thresholds of >90 decibel sound pressure level (dB SPL) at all frequencies (Fig. 1D). In contrast, *Myo15a*^*jd/+*^ and *Myo15a*^*sh2/+*^ heterozygous littermates had normal thresholds (<40 dB SPL) (Fig. 1D). Failure of complementation in *Myo15a*^*jd/sh2*^ mice confirms that the p.D1647G mutation in *Myo15a* is the cause of recessive deafness in the *jordan* pedigree.

All reported mutant *Myo15a* mouse alleles cause profound deafness (MGI:1261811), measured from P14 onwards^21,23,25,39^. ABR showed that several *Myo15a*^*jd/jd*^ mice had residual hearing at 3 months (Fig. 1B, red circles), suggesting a distinct mechanism of hearing loss. We investigated this using longitudinal ABR measurements. At 4 weeks of age, *Myo15a*^*jd/jd*^ mice had moderate hearing loss with broadband click ABR threshold of 53 ± 11 dB SPL, compared to normal hearing *Myo15a*^*+/+*^ (26 ± 6 dB SPL) and *Myo15a*^*+/jd*^ (24 ± 6 dB SPL) littermates (Fig. 1E). The hearing of *Myo15a*^*jd/jd*^ mice progressively worsened, with click ABR thresholds of 53 ± 11, 69 ± 9, 80 ± 9 and 81 ± 8 dB SPL at 4, 6, 9, and 12 weeks, respectively (Fig. 1E, F and Supplementary Fig. 1D–G). Click ABR thresholds for control *Myo15a*^*+/+*^ and *Myo15a*^*jd/+*^ littermates ranged between 22 ± 4 and 26 ± 6 dB SPL, showing no hearing loss with age (Fig. 1E, F and Supplementary Fig. 1D, G). To investigate outer hair cell (OHC) function we measured distortion-product otoacoustic emissions (DPOAEs), and found they were absent in *Myo15a*^*jd/jd*^ mice at 12 weeks, except for frequencies <10 kHz, where they were significantly reduced compared to *Myo15a*^*+/+*^ and *Myo15a*^*+/jd*^ littermates (Supplementary Fig. 1H). The absence of DPOAEs shows that OHC function is impaired in *Myo15a*^*jd/jd*^ mice. As the only known mouse *Myo15a* variant to cause progressive hearing loss, the *jordan* allele is an important model to explore the full spectrum of DFNB3 deafness, which presents heterogeneously as either profound congenital, or progressive hearing loss in humans^16^.

### Stereocilia do not properly elongate in *jordan* mutant hair cells

**Fig. 2:**
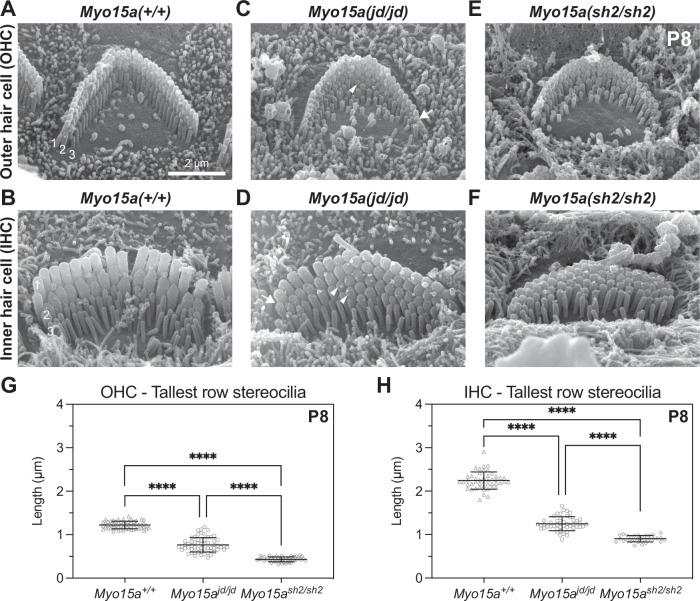
Stereocilia growth is disrupted in *jordan* mutant hair cells. **A–F** Representative SEM images of stereocilia bundles from *Myo15a*^*+/+*^, *Myo15a*^*jd/jd*^, and *Myo15a*^*sh2/sh2*^ OHCs (**A**, **C**, **E**) and IHCs (**B**, **D**, **F**) at P8. In *Myo15a* ^*+/+*^ mice, both IHC and OHC bundles display the characteristic staircase architecture with 3 stereocilia ranks of increasing height (labeled in white font). IHC and OHC bundles from either *Myo15a*^*jd/jd*^ and *Myo15a*^*sh2/sh2*^ mice are shorter in height than the wild-type bundles. *Myo15a*^*jd/jd*^ stereocilia taper in height towards the periphery of the bundle (arrow), and additional stereocilia rows are visible (arrow head). *Myo15a*^*sh2/sh2*^ hair cells also have additional stereocilia rows, but they lack graded thickness and height. **G** Projected heights of tallest (row 1) OHC stereocilia at P8 are 1.2 ± 0.1 µm (*Myo15a*^*+/+*^, 58 stereocilia from 4 mice), 0.8 ± 0.1 µm (*Myo15a*^*jd/jd*^, 60 stereocilia from 4 mice), and 0.4 ± 0.1 µm (*Myo15a*^*sh2/sh2*^, 50 stereocilia from 2 mice). **H** Projected heights of tallest (row 1) IHC stereocilia at P8 are 2.2 ± 0.2 µm (*Myo15a*^*+/+*^, 47 stereocilia from 4 mice), 1.3 ± 0.1 µm (*Myo15a*^*jd/jd*^, 47 stereocilia from 4 mice), and 0.9 ± 0.1 µm (*Myo15a*^*sh2/sh2*^, 30 stereocilia from 2 mice). ****, *P* < 0.0001, Brown–Forsythe and Welch ANOVA with Dunnett’s T3 multiple comparisons test. Representative images are from mid-cochlear turn. Data are mean ± SD. Scale bar, 2 µm.

To investigate the cellular pathology underlying hearing loss in *Myo15a*^*jd/jd*^ mice, we used scanning electron microscopy (SEM) to assess the structure of cochlear hair bundles. In wild-type *Myo15a*^*+/+*^ IHCs and OHCs at P8, stereocilia were graded into a staircase pattern with three distinct rows (Fig. 2A, B). By comparison, bundle heights were reduced in *Myo15a*^*jd/jd*^ littermates, whilst still retaining the staircase architecture (Fig. 2C, D). Quantification of row 1 stereocilia heights showed that *Myo15a*^*jd/jd*^ IHCs and OHCs were significantly shorter than *Myo15a*^*+/+*^ controls (Fig. 2G, H). Furthermore, stereocilia at the lateral edge of *Myo15a*^*jd/jd*^ OHCs, and to lesser extent IHCs, were shorter in height such that the central stereocilia were tallest (Fig. 2C, D). Qualitatively, *Myo15a*^*jd/jd*^ hair cells consistently had 1 or 2 additional rows of stereocilia (Fig. 2C, D) when compared to the three morphologically well-defined rows in *Myo15a*^*+/+*^ IHCs and OHCs (Fig. 2A, B). The additional row phenotype of *Myo15a*^*jd/jd*^ hair cells was similar to *Myo15a*^*sh2/sh2*^ hair cells at P8 (Fig. 2E, F), however *Myo15a*^*jd/jd*^ stereocilia lengths were significantly longer when compared to *Myo15a*^*sh2/sh2*^ hair cells (Fig. 2G, H). We conclude that stereocilia in *Myo15a*^*jd/jd*^ hair cells elongate more than *Myo15a*^*sh2/sh2*^ hair cells, but fail to reach normal wild-type heights. The *Myo15a shaker-2* and *jordan* allelic series shows that mutations in the motor domain led to altered stereocilia heights, and that the increased height in *jordan* hair cells explains why the mice initially have less severe hearing loss than the *shaker-2*.

To further investigate the progressive component of hearing loss, we next examined hair bundle morphology at 12 weeks, when *jordan* mice were profoundly deaf (Fig. 1F). Compared with *Myo15a*^*+/+*^, IHC bundles of *Myo15a*^*jd/jd*^ mice had lost their staircase architecture (Supplementary Fig. 2A) and were still significantly shorter (Supplementary Fig. 2B). Since the staircase was initially present at P8 in *Myo15a*^*jd/jd*^ IHCs (Fig. 2D), these data suggested a postnatal resorption of stereocilia. Consistent with this hypothesis, short stereocilia at the hair bundle periphery were observed at 12 weeks in *Myo15a*^*jd/jd*^ OHCs (Supplementary Fig. 2C, D). These data argue that the progressive hearing phenotype in the *jordan* mouse was due to postnatal stereocilia bundle degradation. Overall, we conclude that the *jordan* allele causes hearing loss by interfering with both initial elongation of stereocilia and their postnatal maintenance. This phenotype was consistent with the *jordan* mutation being within the ATPase motor domain common to both MYO15A-1 and MYO15A-2 isoforms that have independent functions shaping the hair bundle^23^.

### MYO15A traffics the elongation complex in *jordan* hair cells

**Fig. 3:**
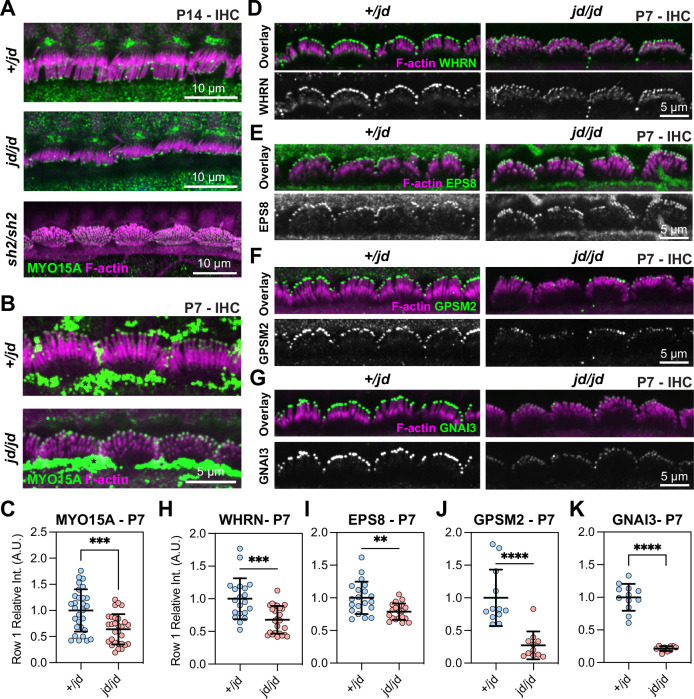
Trafficking of MYO15A and elongation complex in *jordan* hair cells. **A**, **B** Immunofluorescence (IF) confocal images showing anti-MYO15A (green, PB48) in control *Myo15a*^*+/jd*^, *Myo15a*^*sh2/sh2*^, and *Myo15a*^*jd/jd*^ IHCs fixed at P14 (**A**), and *Myo15a*^*+/jd*^ and *Myo15a*^*jd/jd*^ IHCs at P7 (**B**). Phalloidin was used to label F-actin (magenta). Strong extra-cellular PB48 labelling was observed independent of genotype at P7 and thought to be artefactual (asterisk). **C** Quantification of MYO15A antibody (PB48) at the tips of row 1 stereocilia in P7 IHCs. *N* = 29 hair bundles (*+/jd*) and *n* = 29 hair bundles (*jd/jd*). Three independent mice were quantified per condition. **D**–**G** IF confocal images of elongation complex proteins (green) WHRN (**D**), EPS8 (**E**), GPSM2 (**F**) and GNAI3 (**G**) in control *Myo15a*^*+/jd*^ and *Myo15a*^*jd/jd*^ IHCs fixed at P7, overlaid with phalloidin labelled F-actin (magenta). **H**–**K** P7 quantification of IF labelling at row 1 stereocilia tips for WHRN (*+/jd*: *n* = 20, *jd/jd*: *n* = 25) hair bundles (**H**), EPS8 (*+/jd*: *n* = 20, *jd/jd*: *n* = 20) hair bundles (**I**), GPSM2 (*+/jd*: *n* = 12, *jd/jd*: *n* = 11) hair bundles (**J**) and GNAI3 (*+/jd*: *n* = 12, *jd/jd*: *n* = 12) hair bundles (**K**). Two (GPSM2 + GNAI3) and three (WHRN + EPS8) independent mice quantified per condition. Images are representative of data from at least two independent animals per genotype / antibody. Images comparing antibody labelling between *+/jd* and *jd/jd* genotypes are mapped equally. Data are mean ± SD, ****, *P* < 0.0001, ***, *P* < 0.001, **, *P* < 0.01, computed using a Mann-Whitney U-test.

Hair bundle development requires MYO15A-dependent trafficking of the elongation complex (EC) consisting of EPS8, WHRN, GNAI3, and GPSM2. Mutations that prevent MYO15A trafficking (i.e., *shaker-2*) or inactivate individual EC proteins cause a short stereocilia bundle phenotype^27–30,32,34^. Our finding that stereocilia lengths in the *jordan* mouse were only marginally longer than those in the *shaker-2* led us to hypothesize that MYO15A trafficking was defective in *jordan* hair cells. We used the previously validated pan-MYO15A antibody PB48 (Fig. 1A), that binds an epitope common to all isoforms^22,23^, and to the *jordan* mutant, to detect MYO15A in fixed cochleae from *Myo15a*^*jd/jd*^ mutants and *Myo15a*^*+/jd*^ littermates at P14. As expected, in *Myo15a*^*+/jd*^ mice, PB48 labelling was concentrated at the tips of all stereocilia rows in IHCs (Fig. 3A)^24,28,40^. We confirmed that PB48 did not label the short stereocilia of *Myo15a*^*sh2/sh2*^ IHCs at P14 (Fig. 3A), consistent with MYO15A being absent from the bundle^24^. In stark contrast with the *shaker-2*, we observed PB48 labelling at the tips of IHC stereocilia in *Myo15a*^*jd/jd*^ mice at both P7 and P14 (Fig. 3A, B). Although MYO15A still targeted to stereocilia at P7, quantification of normalized PB48 fluorescence at the tips of row 1 stereocilia in *Myo15a*^*jd/jd*^ IHCs revealed a statistically significant 36% reduction compared with *Myo15a*^*+/jd*^ littermates (Fig. 3C). These data suggest that the *jordan* and *shaker-2* mutations affect stereocilia growth via potentially different mechanisms. The *shaker-2* mutation completely blocks MYO15A trafficking, whilst the *jordan* mutation allows MYO15A to traffic at a reduced level that is unable to support normal stereocilia elongation.

We next investigated if the *jordan* mutation interfered with MYO15A-dependent trafficking of the EC proteins, which are necessary for stereocilia growth. To test this, mutant *Myo15a*^*jd/jd*^ and control *Myo15a*^*+/jd*^ littermate P7 cochleae were fixed and labelled with previously validated antibodies to localize WHRN, EPS8, GPSM2, and GNAI3. All of the EC proteins were concentrated at the tips of the tallest stereocilia (row 1) in *Myo15a*^*+/jd*^ control hair cells at P7 (Fig. 3D), in agreement with previous work using wild-type mice^27,30–33^. All four proteins were still qualitatively targeted to the stereocilia tips in mutant *Myo15a*^*jd/jd*^ hair cells at P7 (Fig. 3D–G), however, quantification of WHRN, EPS8, GPSM2, and GNAI3 antibody labelling at the tips of IHC row 1 stereocilia revealed statistically significant reductions in all proteins (Fig. 3H–K). WHRN labelling was reduced by 32% in *Myo15a*^*jd/jd*^ hair cells compared with *Myo15a*^*+/jd*^ littermates (Fig. 3H). Similarly, EPS8 labelling was reduced by 21% in *Myo15a*^*jd/jd*^ hair cells compared with *Myo15a*^*+/jd*^ littermates (Fig. 3I). The signal intensity of GPSM2 and GNAI3 were impacted more severely in *Myo15a*^*jd/jd*^ hair cells, with labelling reduced by 73% and 79% in *Myo15a*^*+/jd*^ littermates, respectively (Fig. 3J, K). When examined in older animals at P14, EPS8, and WHRN were still qualitatively targeted to the tips of *Myo15a*^*jd/jd*^ hair cell stereocilia, however GNAI3 and GPSM2 were substantially reduced compared with *Myo15a*^*+/jd*^ littermates (Supplementary Fig. 3). These data are consistent with MYO15A, EPS8, and WHRN forming a high affinity complex, with GPSM2 and GNAI3 operating as a distinct module^34^. We conclude that the initial defective stereocilia elongation in *jordan* hair cells observed at P7 was not due to the complete disruption of EC trafficking by MYO15A, although EC trafficking was reduced during this early developmental period and significantly disrupted by P14.

### Actin barbed-end capping is unaffected in *jordan* mutant hair cells

**Fig. 4:**
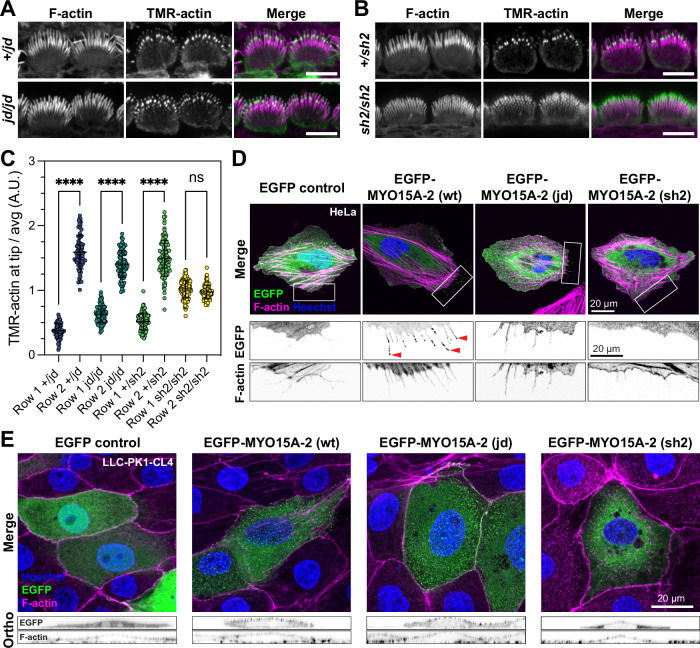
The *jordan* mutation does not disrupt barbed-end capping in stereocilia, but does alter MYO15A trafficking on cellular actin filaments. **A**, **B** Actin barbed-end assay in detergent-permeabilized inner hair cells from mouse cochlear explants acutely isolated at P7. TMR-labelled G-actin (green) was added prior to fixation to identify uncapped barbed ends. Phalloidin labelling of F-actin (magenta) is overlaid. In both *Myo15a*^*jd/jd*^ and littermate *Myo15a*^*+/jd*^ controls, barbed-ends were detected at row 2 stereocilia tips, and at the tips of all stereocilia rows in *Myo15a*^*sh2/sh2*^ hair cells. **C** Quantification of TMR-barbed end incorporation in row 1 and 2 stereocilia (from left to right columns, *n* = 75, 81, 98, 90, 102, 99, 119, 102 stereocilia respectively, 9 hair cells from 3 independent mice per genotype), ****, *P* < 0.0001, one-way ANOVA with Šídák’s multiple comparisons test. **D** HeLa cells were transfected with EGFP-tagged *Myo15a-2* expression constructs or EGFP alone (green) as indicated, fixed and probed with phalloidin (magenta) and Hoechst (blue). Wild-type protein trafficked to filopodia tips (red arrowheads), while *jordan* and *shaker-2* mutants did not. Boxed regions are magnified (inverted grayscale). **E** LLC-PK1-CL4 cells were transfected with EGFP-tagged *Myo15a-2* (green), fixed and labelled with phalloidin (magenta) and Hoechst (blue). The *jordan* mutant concentrated at microvillar tips, similar to wild-type, whereas the *shaker-2* mutant did not. Orthogonal projections are shown (inverted grayscale). Images are representative from at least three independent experiments. Data are mean ± SD. Scale bars, 5 µm (**A**, **B**); 20 µm (**D**, **E**).

The presence of the EC at stereocilia tips of *jordan* hair cells suggested that the proteins known to stimulate growth were present, but insufficient to drive elongation. We considered whether an overall inhibition of actin polymerization was preventing stereocilia elongation in *jordan* hair cells. Capping proteins (CAPZ, TWF2) are present within stereocilia and regulate filament polymerization by blocking barbed-end elongation^41,42^. To detect free barbed ends, we monitored the incorporation of TMR-labelled actin monomers in acutely isolated and detergent permeabilized cochlear explants at P7. In control *Myo15a*^*+/jd*^ inner hair cells (Fig. 4A), TMR-actin was concentrated at row 2 stereocilia tips revealing the presence of uncapped barbed ends, similar to previous reports from wild-type hair cells^43,44^. TMR-actin incorporated at significantly lower amounts at row 1 tips, arguing that barbed ends were inaccessible and capped at that location (Fig. 4A, C). The *jordan* mutation did not alter this distribution of free barbed ends, as we observed a similar incorporation of TMR-actin in *Myo15a*^*jd/jd*^ hair cells compared to controls (Fig. 4A, C). In striking contrast, TMR-actin was incorporated at the tips of all stereocilia rows in *Myo15a*^*sh2/sh2*^ hair cells (Fig. 4B, C), indicating the abnormal presence of barbed ends in row 1 and an overall loss of hair bundle row identity, as previously reported^43,44^. These data suggest that *jordan* hair cells retain the correct identity of row 1 stereocilia up to at least P7, consistent with row 1 stereocilia remaining capped and MYO15A targeting the elongation complex to this location, albeit at lower quantities. These data further argue that inappropriate actin filament capping is unlikely the cause of short stereocilia in *jordan* hair cells. Instead, we hypothesized that a stimulatory factor independent of the elongation complex was missing.

### The *jordan* mutation affects the interaction of MYO15A with actin filaments

The *jordan* missense substitution is in the MYO15A motor domain helix-loop-helix (HLH) motif that forms part of the direct binding interface with the actin filament^37^. We hypothesized that a defect in MYO15A’s interaction with actin might underlie the *jordan* phenotype. To explore this, we examined MYO15A-2 trafficking along filopodia, which are actin-based structures that protrude from the periphery of heterologous cells^24,28^. We focused on MYO15A-2, as it is the isoform responsible for stereocilia growth during development^23^. In transfected HeLa cells, EGFP-tagged wild-type MYO15A-2 accumulated at filopodia tips, indicating robust anterograde myosin movement along the filopodia shaft (Fig. 4D). Discrete puncta of MYO15A-2 were observed along the filopodia shaft, presumably arising from retrograde actin filament treadmilling^28,45,46^. EGFP alone did not accumulate within filopodia, proving this distribution required active myosin motility (Fig. 4D). In cells expressing the MYO15A-2 *jordan* mutant, EGFP was observed diffusely along filopodia shafts and was not concentrated at filopodia tips (Fig. 4D). This was qualitatively similar to cells expressing the MYO15A-2 *shaker-2* mutant (Fig. 4D), which was previously shown to not traffic along filopodia^28^. The inability of the MYO15A-2 *jordan* mutant to traffic within filopodia and accumulate at filopodia tips was curious and contrary to MYO15A protein accumulating at the stereocilia tips of *Myo15a* ^*jd/jd*^ hair cells (Fig. 3A).

Myosins are sensitive to actin filament topology^47–50^ and we hypothesized that filopodia might not contain the appropriate repertoire of actin-binding proteins (ABPs) to support motility. To test this hypothesis, we used the porcine LLC-PK1-CL4 (CL4) epithelial cell line that generates microvilli and is a more accurate model for stereocilia^51^. In CL4 cells transfected with wild-type EGFP-MYO15A-2, EGFP positive puncta localized at the tips of microvilli (Fig. 4E, orthogonal projections). In striking contrast to HeLa cells, the MYO15A-2 *jordan* mutant also concentrated into microvilli and was indistinguishable from the wild-type in CL4 cells (Fig. 4E). No microvillar accumulation of the MYO15A-2 *shaker-2* mutant was observed (Fig. 4E). We conclude that whilst *jordan* and *shaker-2* mutants are both immobile within filopodia, the *jordan* mutant can still actively concentrate in microvilli. These data mimic our findings from hair cells in vivo, and support a change in actin binding as being central to the stereocilia growth defect phenotype observed in *jordan* hair cells.

### The ATPase activity of MYO15A is altered by the *jordan* mutation

**Fig. 5:**
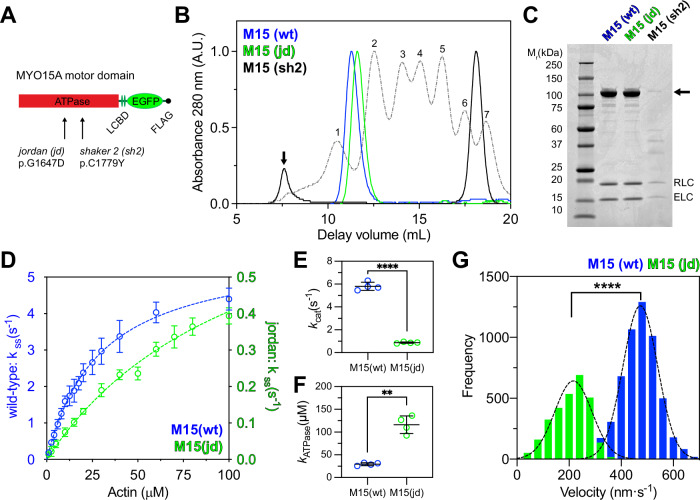
The *jordan* MYO15A motor domain is enzymatically and mechanically active. **A** Cartoon of truncated MYO15A minimal motor domains expressed in *Sf*9 cells, consisting of the ATPase and two light-chain binding domains (LCBD) that bind to ELC and RLC light chains. Residue positions refer to mouse MYO15A-1 (NP_034992.2). **B** Size exclusion chromatography (SEC) analysis of FLAG/IEX purified M15-wt and M15-jd, and of FLAG purified M15-sh2 proteins. Protein calibration standards are shown for comparison (dotted lines); (1) thyroglobulin, (2) ferritin, (3) aldolase, (4) conalbumin, (5) ovalbumin, (6) carbonic anhydrase, (7) ribonuclease A. FLAG purified M15(sh2) was heavily aggregated and analyzed directly by SEC (black line). M15(sh2) eluted close to the void volume (arrow) separate from the FLAG peptide (asterisk). **C** SDS-PAGE analysis of SEC purified motor domain proteins. The motor domain (arrow) co-purifies with RLC and ELC light chains for all variants. M15-sh2 was misfolded and extracted from *Sf*9 cells at low yield. **D** Steady-state ATPase activation of M15-wt and M15-jd motor domains measured using a NADH-coupled assay at 20 ± 0.1 °C. Reactions were performed with [F-actin] as shown. Rectangular hyperbolic fits to averaged data are shown for M15-wt (blue, *k*_cat_ = 5.8 ± 0.2 s^−1^, *k*_ATPase_ = 29.1 ± 2.1 μM, mean ± S.E.M, *n* = 4 experimental determinations) and for M15-jd (green, *k*_cat_ = 0.87 ± 0.04 s^−1^, *k*_ATPase_ = 114.3 ± 8.2 μM, mean ± S.E.M, *n* = 4 experimental determinations). Data points are mean ± SD. **E**, **F** Summary of *k*_cat_ (**E**) and *k*_ATPase_ (**F**) parameters for M15-wt (blue, *n* = 4) and M15-jd (green, *n* = 4). ****, *P* < 0.0001, **, *P* < 0.01, unpaired *t*-test with Welch’s correction. Data points are mean ± SD and from individual experiments in (**D**). **G** Frequency histogram of F-actin velocities in a gliding filament assay at 30 ± 0.1 °C. Gaussian fits (dotted line) are overlaid for M15-wt (473 ± 67 nm⋅s^−1^, *n* = 5449 filaments, mean ± SD) and M15-jd (216 ± 71 nm⋅s^−1^, *n* = 2844 filaments). ****, *P* < 0.0001, unpaired *t*-test with Welch’s correction. All data are from 2 independent protein preparations.

To study the interaction with actin filaments in more detail, we characterized the influence of the *jordan* mutation upon the ATPase mechanochemistry of MYO15A^35,36^. Motor domain proteins along with their associated light chains were expressed in *S. frugiperda* (*Sf*9) insect cells and purified by chromatography (Fig. 5A, C)^35^. In contrast to the *shaker-2* variant (M15-sh2), which aggregated within *Sf*9 cells, both wild-type (M15-wt) and *jordan* (M15-jd) motor domains were soluble. Using size exclusion chromatography (SEC), we found that FLAG purified *shaker-2* motor domain eluted close to the void volume (Fig. 5B). In contrast, the *jordan* motor domain eluted at a similar delay volume to the wild-type (Fig. 5B), which is monomeric^35^. We conclude that the *jordan* variant does not affect folding, whilst the *shaker-2* caused misfolding, consistent with the mutation being within the ATPase globular domain^37^.

To test for differences in enzymatic activity between wild-type (M15-wt) and *jordan* (M15-jd) motor domain variants (Fig. 5A), we measured steady-state ATP hydrolysis using an enzyme-linked NADH assay. The basal ATPase activity (*k*_basal_) of M15-wt was 0.06 ± 0.01 s^−1^, measured without F-actin. The addition of F-actin caused a 97-fold increase in ATPase activity to an extrapolated maximum catalytic rate (*k*_cat_) of 5.8 ± 0.2 s^−1^ (Fig. 5D). Half-maximal activation of the ATPase activity (*K*_ATPase_) was reached at 29.1 ± 2.1 µM F-actin, as previously reported^35,36^. Using identical assay conditions, the basal ATPase rate of M15-jd was unchanged at *k*_basal_ = 0.07 ± 0.01 s^−1^. Strikingly, there was a reduced sevenfold maximal activation to *k*_cat_ = 0.87 ± 0.04 s^−^^1^ (Fig. 5D, note different *y*-axis scale). Furthermore, half-maximal ATPase activation was increased to *K*_ATPase_ = 114.3 ± 8.2 µM actin, signifying a 4-fold reduction in the apparent actin affinity of M15-jd compared to M15-wt in the presence of ATP. These changes were statistically significant (Fig. 5E, F).

The mechanical activity of wild-type and *jordan* motor domains was measured using an in vitro gliding filament assay, where actin filaments are propelled across a microscope cover glass functionalized with motor domain protein^52^. Due to the reduced apparent affinity of M15-jd for actin, we lowered the salt concentration to 10 mM KCl in these assays. Wild-type M15-wt robustly propelled actin filaments at 473 ± 67 nm·s^−1^ (Fig. 5G), consistent with previous data^35^. In contrast, M15-jd moved filaments at 216 ± 71 nm·s^−1^, a twofold reduction from the wild-type velocity (Fig. 5G). Overall, we conclude that whilst the *jordan* mutation caused a significant ATPase defect and decreased the motor domain’s apparent affinity for actin, the motor domain was still mechanically active. Our data establish a functional correlation between motor domain activity and the severity of hearing loss. The *jordan* motor domain retained partial activity consistent with this mutation causing intermediate hearing loss, whilst the *shaker-2* motor domain was misfolded and associated with the most severe phenotype.

### The MYO15A motor domain directly stimulates actin polymerization

Our in vitro analysis of purified MYO15A motor activity was consistent with the *jordan* mutant trafficking the EC to the stereocilia tips in vivo, albeit at a reduced efficiency compared to the wild-type motor. Since stereocilia growth was stunted in *jordan* hair cells, we hypothesized that independent of molecular trafficking, MYO15A might fulfil an additional function critical for elongation. The location of the *jordan* (p.D1647G) missense substitution within the actomyosin binding interface^37^ led us to consider if MYO15A could exert direct control over actin polymerization. This idea was supported by classical biochemical studies of the muscle myosin motor domain (e.g., subfragment-1, S1) that can directly stimulate actin filament assembly in vitro^53–56^.

**Fig. 6:**
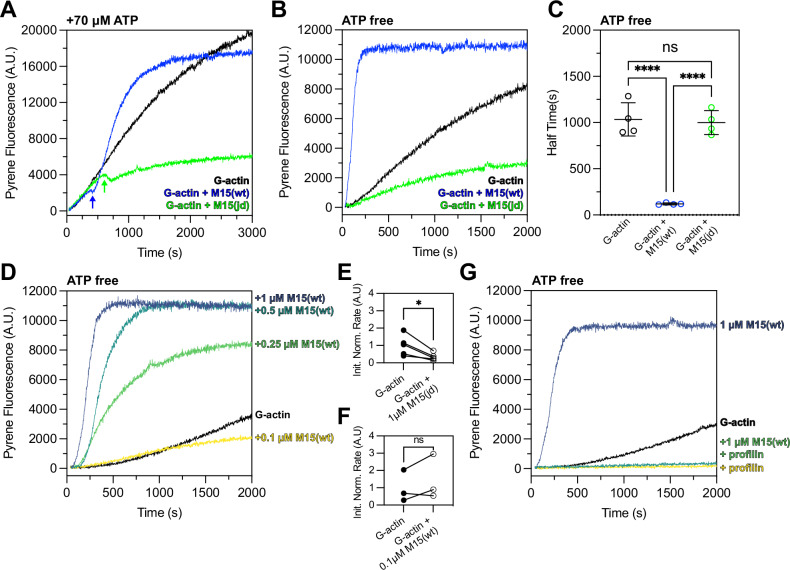
The MYO15A motor domain accelerates actin polymerization in a nucleotide-sensitive manner. **A** Time-course of 2 µM G-actin (10% pyrene labelled) measured in a fluorimeter with polymerization induced by 1× KMEI (50 mM KCl, 1 mM MgCl_2_, 1 mM EGTA, 10 mM imidazole, pH 7.0) at *t* = 0 s (black trace). Addition of either 1 µM M15-wt (blue) or 1 µM M15-jd (green) to the reaction at *t* = 0 s is shown overlaid. M15-wt stimulates polymerization following an inflection point (blue arrow) where free ATP is exhausted. M15-jd does not stimulate polymerization and is instead inhibitory (green arrow). All reactions contain ~70 µM free ATP carried over from the actin storage G-buffer. **B** Experiments from (**A**) repeated using desalted G-actin(ATP) to remove free ATP enforces the strongly bound myosin rigor state. M15-wt immediately increases the rate of actin polymerization (blue), whilst M15-jd does not (green). **C** Quantification of time to reach half-maximal pyrene fluorescence from data in (**B**) (*n* = 4 experimental determinations). Data are mean ± SD. ****,  *P* < 0.0001, one-way ANOVA with Tukey’s multiple comparisons test. **D** Dose-response of M15-wt (0.1–1 µM) upon polymerization of 2 µM desalted G-actin (ATP) induced by KMEI at *t* = 0 s. **E**, **F** Analysis of initial polymerization rates of either 1 µM M15-jd + G-actin (data from **B**, *n* = 5), or 0.1 µM M15-wt + G-actin (data from **D**, *n* = 3), relative to G-actin polymerizing alone. Paired data points are shown with matched G-actin controls measured on the same day. *, *P* < 0.05, paired *t*-test. **G** Profilin inhibits polymerization of desalted G-actin(ATP) stimulated by M15-wt. Reactions were performed as 2 µM G-actin + KMEI (black trace), 2 µM G-actin + 8 µM profilin + KMEI (yellow trace), 2 µM G-actin + 1 µM M15-wt + KMEI (azure trace), 2 µM G-actin + 8 µM profilin + 1 µM M15-wt + KMEI (green trace). All measurements performed at 25 ± 0.1 °C, and are representative of at least 3 experiments, from 2 independent protein preparations.

We tested our hypothesis using pyrene-conjugated globular actin (G-actin) monomers, which increase in fluorescence as they polymerize into filamentous actin (F-actin)^57^. As a control, 2 µM G-actin was polymerized with 1× KMEI (50 mM KCl, 1 mM MgCl_2_, 1 mM EGTA, 10 mM imidazole) while monitoring pyrene fluorescence in the presence of ATP. F-actin assembled with an initial lag phase representing the kinetically unfavourable nucleation step (Fig. 6A, black trace). When the reaction was repeated with 1 µM M15-wt + 2 µM G-actin + KMEI, actin polymerization initially followed the same trajectory (Fig. 6A, blue trace). However, an inflection was observed following a delay of ~400 sec, where the rate of actin polymerization reduced before rapidly increasing and reaching steady-state (Fig. 6A, blue arrow). To test if the increased rate of actin polymerization was sensitive to the *jordan* mutation, we repeated the experiment using 1 µM M15-jd + 2 µM G-actin + KMEI (Fig. 6A, green trace). The actin polymerization rate was again indistinguishable from the control reaction until reaching an inflection point at ~600 s, when the actin polymerization rate subsequently decreased below that of the G-actin + KMEI control (Fig. 6A, green arrow). We conclude that the wild-type MYO15A motor domain stimulated actin polymerization, whilst the deafness-causing *jordan* mutant not only blocked this stimulatory activity, it reduced the overall extent of actin polymerization.

We observed a delay before the actin polymerization rate of either M15-wt or M15-jd deviated from that of the control of G-actin alone + KMEI (Fig. 6A). The delay was longer for M15-jd (~600 s) versus M15-wt (~400 s), and we hypothesized this arose from ATP in the reaction buffer (~70 µM) being hydrolysed faster by M15-wt compared to M15-jd (Fig. 5D). Once ATP was exhausted from the reaction, we reasoned that motor domains would then strongly-bind actin in the nucleotide-free (rigor) or ADP-bound state. To test if the strongly bound rigor state was necessary to stimulate actin polymerization, we repeated the pyrene polymerization assay in the absence of free ATP. To ensure the presence of G-actin(ATP) monomers, G-actin was desalted immediately prior to use to remove free ATP from solution. Using desalted G-actin(ATP) monomers, we measured 2 µM G-actin + KMEI in the absence of free ATP (Fig. 6B) and observed polymerization kinetics comparable to before (Fig. 6A). When the reaction was performed with 1 µM M15-wt + 2 µM G-actin + KMEI (Fig. 6B), pyrene fluorescence saturated with a half-time of 124 ± 12 s, compared with 1091 ± 202 s for G-actin + KMEI alone (Fig. 6C). When the reaction was repeated with 1 µM M15-jd + 2 µM G-actin + KMEI, the overall extent of actin polymerization was reduced compared to G-actin + KMEI (Fig. 6B), and the half-time to saturation did not statistically significantly differ from G-actin + KMEI alone (Fig. 6C). Measurement of pyrene fluorescence within the first 200 s revealed that 1 µM M15-jd slowed the initial rate of actin polymerization compared to the G-actin + KMEI control (Fig. 6E). We conclude that the wild-type MYO15A motor domain strongly accelerated actin filament polymerization in the absence of ATP, implicating the rigor state as critical for this activity. Combined with experiments showing that ADP-bound MYO15A also strongly stimulates polymerization^37^, these data identify the strong actin-binding states as key ATPase intermediates that stimulate actin polymerization. Our data further show that the *jordan* deafness mutation profoundly inhibits the ability of MYO15A to stimulate actin polymerization, linking this activity to hearing loss.

We next explored the dependency of wild-type MYO15A to stimulate actin polymerization in the absence of free ATP. Pyrene actin polymerization assays were performed with 2 µM G-actin + KMEI whilst varying the concentration of M15-wt between 1 µM to 0.1 µM (Fig. 6D). The maximum stimulatory effect was observed at 1 µM M15-wt, and the stimulatory effort was reduced in a dose-dependent manner at 0.5 µM and 0.25 µM M15-wt (Fig. 6D). At 0.1 µM M15-wt, the lowest dose tested, the time-course of pyrene fluorescence was initially similar to the G-actin + KMEI control (Fig. 6D) and had identical initial rates within the first 200 s (Fig. 6F). Over a longer time-course, 0.1 µM M15-wt was inhibitory and reduced pyrene fluorescence below that of the G-actin + KMEI control (Fig. 6D). We conclude that M15-wt can only stimulate actin polymerization at micromolar concentrations that are equimolar with actin. This finding is consistent with the in vivo localization of MYO15A, where it strongly accumulates at the tips of hair cell stereocilia and likely reaches high concentrations. Finally, we tested the ability of the MYO15A motor domain to stimulate polymerization of actin monomers complexed with profilin, a key monomer-binding protein that prevents spontaneous actin nucleation in the cellular cytosol. Pyrene actin assays performed with 2 µM G-actin + 8 µM profilin + KMEI confirmed that profilin inhibited spontaneous actin polymerization (Fig. 6G, yellow trace). The time-course of pyrene fluorescence was indistinguishable when this assay was repeated with 1 µM M15-wt included (Fig. 6G, green trace), demonstrating that the MYO15A motor domain can accelerate polymerization of actin monomers, but not of profilin-bound actin monomers.

### The *jordan* deafness mutation inhibits de novo nucleation of actin filaments

**Fig. 7:**
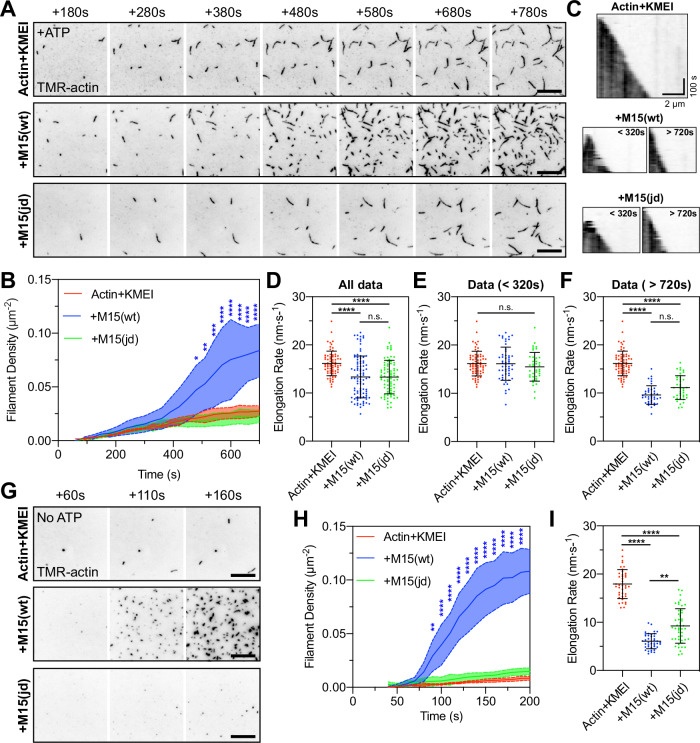
The MYO15A motor domain nucleates actin filaments de novo. **A** TIRFM visualization of actin filaments polymerizing on PEG-biotin-NeutrAvidin functionalized cover glass. Polymerization of 1 µM G-actin (20% TMR + 10% biotin labelled) was induced by KMEI (50 mM KCl, 1 mM MgCl_2_, 1 mM EGTA, 10 mM imidazole, pH 7.0) at *t* = 0 s, in the presence of 25 µM ATP. Representative time-lapses shown for: 1 µM G-actin (top), 1 µM G-actin + 1 µM M15(wt) (middle), and 1 µM G-actin + 1 µM mutant M15(jd) (bottom). **B** Quantification of actin filament density shows delayed nucleation activity of MYO15A in the presence of ATP. *n* = 3 independent determinations. **C** Kymographs of actin filament elongation. **D** Barbed-end elongation rates for G-actin + KMEI (red, *n* = 69 filaments), G-actin + M15(wt) (blue, *n* = 94), G-actin + M15(jd) (green, *n* = 80). **E** Elongation rate data (from **D**) re-binned before nucleation, G-actin alone (*n* = 69 filaments), G-actin + M15(wt) (*n* = 54), G-actin + M15(jd) (*n* = 40). **F** Elongation rate data (from **D**) re-binned after nucleation, G-actin alone (*n* = 69 filaments), G-actin + M15(wt) (*n* = 40), G-actin + M15(jd) (*n* = 40). The G-actin + KMEI control data set (from **D**) is reproduced identically as a comparator in (**E**, **F**). **G** Time-lapse of actin filament polymerization induced by KMEI at *t* = 0 s, with no ATP in solution. G-actin (ATP) monomers were prepared by desalting into ATP-free G-buffer. **H** Actin filament density shows nucleation activity of MYO15A is accelerated in the absence of ATP. G-actin + KMEI (*n* = 4 determinations), G-actin + M15(wt) (*n* = 5), G-actin + M15(jd) (*n* = 5). **I** Barbed-end filament rates in the absence of free ATP. Reaction deadtimes were typically 50 s and included in quantification. TIRFM images are shown as inverted grayscale. G-actin + KMEI (*n* = 40 filaments), G-actin + M15(wt) (*n* = 40), G-actin + M15(jd) (*n* = 47). All data are plotted as mean ± SD. Statistics were computed using two-way ANOVA with Dunnett’s multiple comparisons test (**B**, **H**), and one-way ANOVA (Kruskal–Wallis) with Dunn’s multiple comparisons test (**D**, **E**, **F**, **I**). Statistical significance is denoted by ****, *P* < 0.0001, ***, *P* < 0.001, **, *P* < 0.01. Scale bars are 10 µm (**A**, **G**). Data are from 3 to 5 experimental determinations (**A**–**F**), and 4–5 experimental determinations (**G**–**I**), using 2 independent protein preparations.

To directly visualize how the MYO15A motor domain accelerates polymerization, we performed actin polymerization assays using total internal reflection fluorescence microscopy (TIRFM) where the elongation of individual filaments can be tracked^58^. A control time-lapse of 1 µM G-actin + KMEI revealed a slow rate of filament nucleation, with short polymers attaching to surface and elongating (Supplementary Movie 1 and Fig. 7A, top row). We repeated the experiment with 1 µM M15-wt and observed a burst in filament density (Supplementary Movie 1 and Fig. 7A, middle row) that was statistically significant at 480 s when compared to the actin-alone control (Fig. 7B). Similar to pyrene-actin assays, we suspected this increased rate of actin filament nucleation was only observed once free ATP (initially ~50 µM) had been hydrolysed and myosin heads entered strongly bound states (either ADP-bound or apo). When polymerization experiments were repeated with 1 µM M15-jd (Supplementary Movie 1 and Fig. 7A, bottom row), there was no increase in actin filament density above the actin-alone control (Fig. 7C). These data show that the wild-type MYO15A motor domain increased the rate of actin filament nucleation, whereas the *jordan* mutant could not. Using kymographs to track filament barbed ends, we further found that MYO15A influenced actin filament elongation rates. In control experiments, barbed ends elongated at 16.2 ± 2.6 nm·s^−1^ (Fig. 7C, D), consistent with previous data using TMR-conjugated G-actin on Cys374^59^. Elongation rates were significantly reduced by the addition of either M15-wt or M15-jd, to 13.3 ± 4.3 nm·s^−1^ and 13.4 ± 3.5 nm·s^−1^, respectively (Fig. 7C, D). The decrease in elongation rate was dependent upon timing of the filament burst (Fig. 7B). When data were binned as pre-burst (<320 s), the addition of wild-type or *jordan* motor domains did not significantly alter elongation rates compared to the control (Fig. 7E). In contrast, the presence of either wild-type or *jordan* motor domain post-burst (>720 s) both significantly, and equally, reduced elongation rates below the actin alone control (Fig. 7F).

We next considered whether the production of short filaments was caused by MYO15A directly driving actin filament nucleation, or if it was due to MYO15A producing new barbed ends by severing actin filaments that nucleated spontaneously. To test this hypothesis, we repeated our experiments in the absence of free ATP to force the motor domain into rigor binding and accelerate polymerization (Fig. 6B). Free ATP was desalted from 1 µM G-actin(ATP) monomers as before, and this did not affect barbed-end elongated rates when polymerized by KMEI (Supplementary Movie 2 and Fig. 7D, I). When 1 µM M15-wt was included in the reaction, there was a potent nucleation of short actin filaments observed within 90 s (Supplementary Movie 2 and Fig. 7G, H), confirming acceleration of nucleation when the motor domain was forced into rigor. Under identical ATP-free conditions, 1 µM M15-jd did not nucleate additional filaments compared with the G-actin + KMEI control (Supplementary Movie 2 and Fig. 7G, H). Both M15-wt and M15-jd motor domains significantly reduced barbed-end elongation rates compared to G-actin + KMEI alone (Fig. 7I). We conclude that the MYO15A motor domain exerts multiple direct effects on actin polymerization, including catalyzing de novo filament nucleation and reducing barbed-end elongation rates in a nucleotide-dependent fashion. Our data show that MYO15A-induced actin nucleation is a key process targeted by the *jordan* deafness mutation, and argue that this activity is critical for stereocilia elongation and hearing.

## Discussion

Plasticity of the stereocilia actin core is central to hair bundle development, mechano-sensitivity and hearing. In the currently held model of stereocilia growth, MYO15A traffics along stereocilia actin filaments and delivers EC proteins to the tip compartment where they are hypothesized to control actin filament length. The requirement for these molecules in stereocilia growth is well established, but how MYO15A and EC molecules actually regulate actin polymerization is unknown. In the present study, we show that MYO15A can directly stimulate actin filament nucleation in vitro, and that nucleation is inhibited by the *jordan* (p.D1647G) mutation that stunts stereocilia growth and causes hearing loss in vivo. We propose that in addition to its key activity trafficking the EC, MYO15A directly catalyzes actin filament nucleation at the growing stereocilia tips, and that both EC activity and nucleation processes combined are required for normal stereocilia growth and hearing. More broadly, our work argues that myosin-catalyzed nucleation can influence actin network architecture within cells, and that this mechanism is disrupted during the pathogenesis of cytoskeletal diseases, such as hearing loss.

The MYO15A motor domain exerted multiple effects upon actin filament polymerization in vitro. Using purified proteins and reconstituted assays, the motor domain: (1) nucleated actin filaments de novo, and (2) slowed the barbed-end elongation rate of existing actin filaments. No additional proteins were required for these effects, demonstrating that these properties were intrinsic to the purified actomyosin system. The *jordan* mutation completely blocked MYO15A-induced actin filament nucleation, and whilst the apparent affinity for actin was reduced, filament elongation rates were comparable between *jordan* and wild-type MYO15A. We conclude that MYO15A’s nucleation activity is the key polymerization effect disrupted by the *jordan* deafness mutation in vitro. Similar to MYO15A, muscle myosin was shown in classic biochemical experiments to stimulate actin filament nucleation in vitro^53–56^, however a cellular function for this in muscle has not been reported. The MYO15A motor domain shares a common structural fold with muscle myosin^37^, and whilst the mechanism of muscle nucleation remains controversial^54^, we believe—by homology to muscle—that MYO15A nucleates actin through stabilizing inter-subunit contacts that establish the protofilament^60,61^. In a previous study^37^, the MYO15A motor domain was further shown to influence structural plasticity within the DNaseI-binding loop (D-loop), a sub-domain of actin that regulates the monomer to polymer transition^62^. Many myosin motor domains share significant homology surrounding the acidic residue mutated in the *jordan* allele (p.D1647, Supplementary Fig. 1C), and it is exciting to consider if other myosins critical for hearing, including MYO1C, MYO3A, MYO6, and MYO7A^63–69^, can directly regulate actin nucleation as part of their cellular function in hair cells. Our findings expand upon the existing repertoire of myosin motors to control actin polymerization by recruiting nucleation-promoting factors^70,71^, and through generating mechanical force. Clustering of myosin ATPase domains at the plasma-membrane is sufficient to stimulate filopodia production in a variety of cell lines, implicating myosin ATPase activity, and thus force production at the membrane^72^. Modifying ATPase activity also alters filopodia growth induced by MYO3A, further arguing that force production is critical^73,74^. Whether actin-nucleation by myosin ATPase domains can also contribute to filopodia assembly, and to what extent, is an important question to be addressed in future work. In summary, our data shows that nucleation is a shared function of at least some members of the myosin superfamily, and argues that in addition to generating force, they potentially influence cytoskeletal plasticity by catalyzing actin nucleation.

Actin nucleation by MYO15A is distinct from mechanisms of other cellular nucleation factors, such as formins, Arp2/3, Spire, Leiomodin and Cobl^75–81^. MYO15A-driven nucleation was nucleotide-sensitive, and strongly promoted nucleation under rigor (nucleotide-free) conditions, or when bound to ADP^37^ as shown in our companion manuscript; both conditions populate the motor domain into strong actin-binding states^36^. The presence of ATP in assays prevented nucleation (Figs. 6A and 7A, B < 320 s) and we hypothesize this is due to the motor domain populating weakly actin-bound states^36^. Whilst the stereocilia tip compartment is not expected to be nucleotide-free, the exact concentrations of ATP and ADP are unknown. A creatine kinase ATP regeneration system is present in avian hair cells^82^, but the components of this system are not concentrated in murine stereocilia^83^, such that ADP could potentially accumulate at steady-state. The nucleotide-sensing properties of MYO15A may thus provide a mechanism to couple actin polymerization with the local ratio of ATP/ADP, and we speculate this could be the basis of a tuning mechanism where nucleotide concentrations control the height of developing stereocilia. We further speculate that alterations in the ATP/ADP ratio could be influenced by the activity of plasma membrane Ca^2+^ ATPase pumps (PMCA2) that are highly enriched in stereocilia^84^. Our work aligns with recent research demonstrating that the mechanical stiffness of actin filaments is also contingent on nucleotide status, highlighting that nucleotide-dependent modifications may exert more extensive influence over actomyosin networks than previously acknowledged^85^.

MYO15A-driven nucleation in vitro was only observed at micromolar concentrations that were approximately equimolar with actin, consistent with previous reports of muscle myosin nucleation^55,60^. MYO15A at 100 nM did not nucleate in pyrene actin assays and instead exerted a weakly inhibitory effect, potentially by reducing the barbed-end elongation rate. These findings are in contrast with other cellular actin nucleators, such as DIAPH1, that are potent at nanomolar concentrations^86^. The micromolar concentration required for MYO15A-driven nucleation potentially restricts nucleation activity to the stereocilia tip compartment, where MYO15A actively accumulates in vivo^23,24,40^. Further evidence supporting the feasibility of MYO15A-induced nucleation comes from recent studies demonstrating that MYO15A, in conjunction with EC proteins, undergo liquid-liquid phase separation (LLPS) to form biomolecular condensates that may contribute to formation of the stereocilia tip density^87,88^. Incorporation of MYO15A into a liquid condensate would not only sustain high protein concentrations at the stereocilia tip, but also raise the possibility that condensed MYO15A and EC proteins could form a membrane-less reaction compartment optimized to catalyze actin nucleation. The activity of MYO15A within this condensate could be regulated through interactions with EC proteins in addition to molecular crowding effects. Furthermore, MYO15A may itself anchor the biomolecular condensate at the site of actin polymerization and stereocilia growth via its C-terminal FERM domains binding to phosphoinositol-4,5-bisphosphate (PIP_2_) that are highly enriched at stereocilia tips^89,90^. Understanding the role of biomolecular condensation in regulating MYO15A activity and actin polymerization is an important area of future research.

Actin monomers are typically complexed with monomer-binding proteins, such as thymosin β4 and profilin, that prevent spontaneous actin polymerization in the cytosol. We found that MYO15A was unable to nucleate profilin-actin in vitro, suggesting additional regulatory mechanisms can influence MYO15A-driven nucleation in hair cells. Other actin nucleators, such as formins, can selectively interact with profilin isoforms allowing them to polymerize specific pools of actin monomers^91^. Our experiments used profilin from *Saccharomyces pombe* (Pfy1) and more work is needed to determine how the mammalian profilin paralogs (PFN1, PFN2, PFN3, PFN4) might inhibit, or potentiate actin nucleation by MYO15A in the cytosol versus stereocilia compartment. PFN2 has been detected in purified hair bundles by mass-spectrometry^92^, however the presence of any monomer-binding proteins have yet to be conclusively demonstrated at the stereocilia tip compartment. The changing availability and identity of monomer-binding proteins during hair cell development and maturation could add additional layers of regulation to MYO15A’s activity. In support of this model, the abundance of thymosin β4 (TMSB4X) decreases concomitantly with hair cell differentiation and is proposed to free actin monomers for stereocilia elongation as nascent hair cells start assembling the hair bundle^93^. We speculate that the reduction in the abundance of monomer-binding proteins is a permissive condition that triggers MYO15A’s nucleation activity. Future exploration of the actin-binding protein regulatory landscape will help define the activity of MYO15A within the context of stereocilia development.

The relative contributions of MYO15A nucleation versus EC activity towards stimulating stereocilia elongation remain unresolved. The EC proteins are independently critical for stereocilia growth and require MYO15A to be properly trafficked to the stereocilia tip^28–32,34^; these observations have supported a model where MYO15A’s function is to traffic molecules required for elongation. The *jordan* mouse allowed us to hypothesize that EC proteins were insufficient to drive normal stereocilia elongation, as these proteins still accumulated in short stereocilia at P7. Combined with our finding that the *jordan* mutation completely blocked MYO15A-catalyzed actin nucleation, these data argue that nucleation is an important component of the stereocilia elongation mechanism. However, the *jordan* mutation additionally slowed in vitro gliding filament velocities by ~50% compared to the wild-type ATPase activity, and this was consistent with reduced quantities of MYO15A and EC proteins quantified at the stereocilia tips at P7. The reduction was modest for EPS8 and WHRN, but particularly evident for GNAI3 and GPSM2. We therefore cannot exclude that defective stereocilia growth is caused, in part, by the reduction in MYO15A and EC trafficking in *jordan* mice. The total amount of MYO15A at stereocilia tips has been correlated with stereocilia lengths^24,40^, however this finding convolves separate MYO15A-1 and MYO15A-2 isoforms that distribute to distinct stereocilia rows^23^, and does not represent a gradient of a single MYO15A species. Over-expression of the developmental MYO15A-2 isoform via biolistic transfection in vitro can drive stereocilia elongation in *shaker-2* hair cells, but there are conflicting data as to whether this results in over-elongation^28,30^. Reduced accumulation of MYO15A is reported in the short stereocilia of *Whrn*, *Eps8*, *Gpsm2*, and *Gnai3* mouse mutants in vivo, in parallel with reduced accumulations of EC proteins^30,32,34^. Whilst these findings do argue for a reduction in EC protein concentration interfering with stereocilia elongation, our results indicate that alterations of MYO15A concentration will change EC trafficking and MYO15A-driven nucleation synchronously. Future work is now needed to separate the trafficking and nucleation activities of MYO15A in hair cells; this will be complicated by both functions depending upon the identical actin - binding interface, thus making them difficult to perturb independently. In summary, our results challenge the conventional understanding of EC sufficiency for driving stereocilia elongation, and argue that the EC proteins are unable to drive full stereocilia elongation without MYO15A-catalyzed nucleation.

The *jordan* mutation allowed new insight into how MYO15A traffics along actin filaments. MYO15A-2 was previously reported to concentrate at the tips of actin-based filopodia^24,28^ and our results confirm this in HeLa epithelial cells. The MYO15A-2 *jordan* mutant strikingly failed to exhibit such localization in HeLa cells, yet conversely in the LLC-PK1-CL4 renal epithelial cell line was able to robustly localize to the tips of microvilli, similar to our observations in hair cells. This intriguing discrepancy between filopodia and microvilli/hair cells can be potentially explained by variations in ABPs and actin filament topology present in filopodia versus microvilli. Myosins are sensitive to their actin filament substrate and MYO15A may be optimized to interact with the more complex ABP environment present within microvilli and stereocilia^47,50,94–96^. In this model, microvilli bundled actin filaments would support some motility of the *jordan* mutant, but filopodial actin filaments could not. This may be compounded by differing rates of actin treadmilling, where a barbed-end myosin motor must overcome retrograde flow to reach the filopodia tip, similar to the analogy of successfully running up a down-escalator. The treadmilling rate in filopodia is 2–3× faster than in CL4 microvilli^97,98^. We speculate that the *jordan* mutant MYO15A might not overcome retrograde flow in filopodia, but has sufficient velocity to do so in microvilli, and by extension stereocilia that do not treadmill. Future work is needed to explore these intriguing hypotheses.

The ability of MYO15A to nucleate actin filaments raises questions concerning the stability and plasticity of the stereocilia cytoskeleton. Stereocilia actin filaments are unidirectionally polarized with their fast-growing barbed ends orientated towards the tip compartment^7,8^. The core actin filaments are relatively stable and do not treadmill like other actin-based structures, such as microvilli and filopodia^97,98^; the tip compartment is highly plastic however, and monomers are added here during developmental elongation and continue to turnover throughout adult life^10,12,13,43^. Why might a nucleator be needed to elongate pre-existing filaments, given the availability of barbed ends at the stereocilia tips? One possible explanation is that stereocilia elongation occurs through direct end-to-end annealing of short actin polymers, rather than through addition of individual actin monomers at the barbed end. Filament annealing is an intrinsic property of actin; short filaments can self-anneal into longer filaments in vitro^99–101^, and more recent in vitro studies have detected filament extension through incorporation of short polymers, in addition to the dominant mode of monomer addition^102,103^. Endocytosis in yeast is proposed to use end-to-end actin filament annealing rather than polymerization of monomers, providing evidence of this phenomenon in vivo^104^. We did not observe filament annealing in our single-filament TIRFM recordings, likely because actin filaments were immobilized on the mPEG-NeutrAvidin functionalized surface, restricting the chances of a diffusional encounter. We speculate that the extension and remodelling of the stereocilia actin core using short polymer annealing may allow for more rapid changes in stereocilia architecture needed to continuously maintain the sensitivity of the hair bundle and MET machinery^6,26^.

As a potent nucleator of actin filaments, we infer that the motor domain of MYO15A must be tightly regulated in hair cells. There is extensive evidence for intramolecular regulation throughout the myosin superfamily^105^. For example, myosin 5 (MYO5A) exists in an autoinhibited conformation where the globular tail domain binds and inhibits motor domain activity; binding of melanophilin (MLPH) to MYO5A releases this autoinhibition to activate the motor domain^106–110^. Similar autoinhibitory regulation mechanisms have been described for other myosins that contain MyTH4-FERM domains, comparable to MYO15A^111–115^. We hypothesize that MYO15A (Fig. 1A) is similarly auto-inhibited by its tail MyTH4-FERM domains and that one function of the EC is to shift the equilibrium of MYO15A between active and inhibited states. Whilst speculative, this may explain why *Myo15a* mutant alleles, such as *shaker-2* and *jordan*, exhibit shorter and additional stereocilia row phenotypes shared with *Whrn*, *Eps8*, *Gpsm2*, and *Gnai3* mouse mutants^27,28,30,32–34^. In addition to their own specific functions, the loss of any individual EC protein would be predicted to disrupt the regulatory complex controlling MYO15A and its critical activities of driving motility and nucleation in stereocilia. Consistent with this, mutant alleles of EC proteins reduce the accumulation of MYO15A concentrating at the stereocilia tip compartment, potentially signifying a difference in myosin activity^30,32,34^. More work is needed to resolve the basic properties of EC proteins in complex with MYO15A, and how they might regulate actin polymerization. Dissecting the mechanisms of intramolecular regulation will likely also help explain how different MYO15A isoforms independently exert their effects upon hair bundle architecture^23^. MYO15A-1 and MYO15A-2 possess an identical motor domain competent to generate force and regulate actin polymerization, but differ by the addition of a 133 kDa N-terminal domain (Fig. 1A). How the N-terminal domain affects the actin nucleation activity of the motor domain is an important future question and will help uncover how MYO15A-1 selectively controls actin polymerization in shorter stereocilia rows with active MET^23^. In conclusion, we reveal a new molecular function for MYO15A and argue that not only does myosin-based nucleation of actin filaments contribute towards the establishment of stereocilia architecture, but that defective actin nucleation by MYO15A is a central molecular pathology underlying human hereditary hearing loss, DFNB3.

## Methods

### Mice

Pedigree MPC190 was identified from a phenotype-driven mutagenesis screen undertaken at the MRC Harwell Institute^38^. Briefly, *N*-ethyl-*N*-nitrosourea (ENU) mutagenized C57BL/6J males were mated with wild-type ‘sighted C3H’ (C3H.Pde6b^+^) females. Resulting G_1_ males were crossed with C3H.Pde6b^+^ females to produce G_2_ females, which were screened for the *Cdh23*^*ahl*^ allele. *Cdh23*^*+/+*^ G_2_ females were backcrossed to their G_1_ fathers to generate recessive G_3_ pedigrees, which entered a longitudinal phenotyping pipeline that included click box and ABR tests to assess auditory function^116^. DNA from mice exhibiting hearing loss, and normal hearing pedigree mates, was prepared from ear biopsies and used for linkage mapping utilizing the Illumina GoldenGate Mouse Medium Density Linkage Panel (Gen-Probe Life Sciences Ltd, UK), which identified a critical interval on chromosome 11. DNA was extracted from mouse MPC190/2.18a and subject to whole genome sequencing employing the Illumina HiSeq platform (Oxford Genomics Centre, Wellcome Trust Centre for Human Genetics). Subsequent alignment to the reference genome (NCBIM38/mm10) identified a homozygous, non-synonymous coding lesion in the *Myo15a* gene.

At the MRC Harwell Institute, mice were housed and maintained at the Mary Lyon Centre under conditions outlined in the Home Office Code of Practice, with all animal procedures licenced by the Home Office under the Animals (Scientific Procedures) Act 1986, UK and approved by the local Ethical Review Committee (PBF9BD884 to MRB). At MRC Harwell, *jordan* mice were crossed to C57BL/6N (*Cdh23*^*753A>G*^) ‘repaired’ mice^117^ until congenic. Concurrently, *jordan* mice were imported to the NIH and the University of Florida (UF) and maintained on a ‘sightless C3H’ (C3H.Pde6b^rd1^) background. Animal procedures were approved the Institutional Animal Care and Use Committees (IACUC) at UF (#201910739 to JEB) and at the NIH (#1263-15 to TBF). All mice were kept on a 12-h light, 12-h dark cycle. Genomic DNA from mouse tail biopsies was used as template in a PCR reaction with primers (5′-CAGGAGGAGTACATCCGGG-3′, 5′-AGACCACAGAAGTATCTGGGTCTT-3′). The resulting 161 bp amplicon was analyzed by *MlsI* endonuclease digestion. Resulting restriction fragments lengths unambiguously detected wild-type (161 bp) and mutant (116 bp + 5 bp) alleles.

### Auditory phenotyping and behavioral testing

At the MRC Harwell Institute, Auditory Brainstem Response (ABR) and DPOAEs were recorded from *jordan* mice, following a previously described protocol^118^. Briefly, mice were anesthetised via intraperitoneal (IP) injection with ketamine hydrochloride (100 mg⋅kg^−1^) and xylazine (10 mg⋅kg^−1^). Anesthetised mice were placed inside a sound-attenuated chamber (ETS-Lindgren) and recording electrodes (Grass Telefactor F-E2-12) placed sub-dermally over the vertex (active), right mastoid (reference), and left flank (ground). ABR traces were collected using TDT system III hardware and BioSig software (Tucker Davis Technology). Stimuli were presented free field from an ES1 transducer (TDT) calibrated at a distance of 1 cm from the right ear. Stimuli consisted of 0.1 ms broadband clicks or 7 ms tone-bursts at 8, 16, and 32 kHz. Each stimulus was presented at a maximum 90 dB SPL, followed by decreasing steps of 5–10 dB SPL until no replicable response peaks were observed. ABR operators were blind to genotype. DPOAEs were recorded as a terminal procedure in 12 weeks old mice, as described^118^. Mice were anesthetised with a modified ketamine/xylazine solution (see ABR), with the addition of acepromazine (2 mg⋅ml^−1^, 8% v/v). Anesthetised mice had a section of the pinna removed to access the external auditory meatus. Mice were placed inside a sound-attenuated chamber (ETS Lindgren), and the DPOAE probe assembly was inserted into the ear canal. In-ear calibration was performed before each test. An ER10B+ low-noise probe microphone (Etymotic Research) was used to measure DPOAEs near the tympanic membrane. Tone stimuli were presented via separate MF1 (Tucker Davis Technology) speakers, with f1 and f2 at a ratio of f2/f1 = 1.2 (L1 = 65 dB SPL, L2 = 55 dB SPL). The f1 and f2 tones were presented continuously in specific tone-bursts between 8 and 32 kHz.

At the NIDCD, ABRs from *jordan* and *shaker-2* complementation tests were recorded following a previously described protocol^119^. Briefly, mice were anesthetised via IP injection with ketamine (56 mg⋅kg^−1^) and Dexdomitor (0.375 mg⋅kg^−1^). Body temperature was maintained on a 37 C heating plate. ABR signals were recorded in a closed-field configuration using TDT hardware (RZ6 processor and MF1 speakers) and BioSigRZ software (Tucker Davis Technologies) in a sound-proof booth (Acoustic Systems). Sub-dermal needle electrodes were placed at the cranial vertex and below each pinna. Responses to 3 ms tone-bursts at 8, 16, and 32 kHz were initially presented at 80 dB SPL and then decreasing in 10–20 dB intervals to determine the level required for a reproducible response. Close to threshold, the stimulus was reduced in 5–10 dB steps until a response was no longer reproducibly detected. If there was no response at 80 dB the stimulus was increased to 90 dB SPL, and if no reproducible response was detected, the threshold was designated as 100 dB for subsequent analysis.

### Scanning electron microscopy

Inner ears were dissected and fixed overnight at 4 °C in 0.1 M phosphate buffer, 2.5% glutaraldehyde (Sigma-Aldrich). Post-fixed ears were decalcified in 4.3% EDTA in phosphate buffer at 4 °C, before dissecting out the sensory epithelium. Samples were processed with alternating 1% osmium tetroxide (Agar Scientific) in 0.1 M sodium cacodylate (Sigma-Aldrich) and 1% thiocarbohydrazide (Sigma-Aldrich) treatments. Osmicated samples were dehydrated in graded ethanol concentrations (25% to 100%) at 4 °C and stored in 100% acetone until critical point drying with liquid CO_2_ (EM CPD300, Leica Microsystems Ltd.). Samples were mounted onto stubs using silver paint (Agar Scientific), sputter-coated with platinum (Q150R S, Quorum Technologies) and visualized with a scanning electron microscope (JSM-6010LV, JEOL).

For morphometric analyses, image pairs from the middle cochlear turn IHC and OHC bundles were captured with a 5° tilt angle difference at a constant working distance of 20 µm. Stereocilia from at least two bundles per animal were measured using ImageJ (https://imagej.nih.gov), with a minimum of 14 stereocilia for each condition. Length measurements were taken from the rear aspect of the hair bundle, so that the length of the tallest stereocilia (row 1) could be measured from the cuticular plate insertion to the tip. Estimates for actual stereocilia length were calculated using a pseudo-eucentric tilting approach^120^. A single measure *x*_1_ (length of stereocilium) was taken from the first image and measured again (*x*_2_) on the corresponding 5°-tilted image pair. Perpendicular counter-measures (*y*_1_ and *y*_2_), matched to (*x*_1_ and *x*_2_) were also recorded. Equation 1 was used to estimate uncertainty (ζ) due to plane rotation. Using the uncertainty estimate (*z*) from Eq. 1, every pair of tilted measures (*x*_1_ and *x*_2_) was processed using Eq. 2 to obtain a close approximation (*ξ*) of the true length of each stereocilia measured.

1 $$z=\frac{(\Delta y)\cos \varDelta \phi+\left(\frac{2{y}_{1}({y}_{1}-\Delta y)}{d}\right)\sin \Delta \phi }{\left(1+\frac{{y}_{1}({y}_{1}-\Delta y)}{{d}^{2}}\right)\sin (2\Delta \phi )+\left(\frac{\Delta y}{d}\right)\cos (2\Delta \phi )}$$

2 $$\xi=\frac{2d-2z\,\cos (\Delta \phi )}{\frac{d}{{x}_{1}}+\frac{d}{{x}_{2}}}$$

Where: ${{{z}}}$ = uncertainty estimate, $\xi$ = estimate of true size of structure of interest, ${x}_{{1,2}}$ = tilted paired-measures of structure of interest, ${y}_{{1,2}}$ = perpendicular counter-measures to measures ${x}_{{1,2}}$, $\Delta y$ = arithmetic difference of counter-measures ${y}_{1}$ and ${y}_{2}$, $\varDelta \phi$ = tilting angle (5°), $d$ = working distance (20 µm).

In addition, low-magnification (1500–2000×) fields of mid-turn sensory epithelium were imaged and the OHC bundles counted and visually categorized as either: ‘Intact’, ‘Abnormal / Damaged’ where the bundle had an abnormal shape or was missing stereocilia, or ‘Missing Bundle’ where a cuticular plate was observed with no stereocilia. Cochleae from four different animals per genotype were examined, with a total of 1344 wild-type and 972 *jordan* OHC bundles.

### Whole mount immunofluorescence and confocal microscopy

Mouse inner ears were fixed in 4% paraformaldehyde (EMS Diasum) in PBS for 2 h at room temperature, washed in PBS, and then micro-dissected to isolate the cochlear sensory epithelium. Samples were permeabilized in 0.5% (v/v) Triton X-100 in PBS for 30 min at RT, followed by blocking in 5% normal goat serum (Sigma-Aldrich), 2% bovine serum albumin (Fraction V, Roche) in PBS for 1 h at RT. Primary antibodies were diluted in blocking solution and incubated with samples at 4 °C overnight. Primary IgG antibodies used were: PB48 rabbit anti-MYO15A^22^, HL5136 rabbit anti-WHRN^28^, mouse anti-EPS8 (#610143, BD Biosciences), rabbit anti-GPSM2^121^ and rabbit anti-GNAI3 (#G4040, Sigma-Aldrich). After washing in PBS, samples were labelled with Alexa Fluor 488 conjugated anti-IgG secondary antibodies (Life Technologies) diluted in blocking solution for 1 h at RT. Samples were co-labelled with rhodamine phalloidin (Life Technologies) and mounted with high-precision #1.5 cover glass (Thorlabs) using Prolong Gold (Life Technologies). Images were captured using either an inverted microscope with 63× objective (1.4 NA, plan apochromat, Zeiss) and a laser scanning confocal microscope (LSM780, Zeiss), or an inverted microscope (Nikon Ti2-E) with an 100× oil objective (CFI Apochromat TIRF, 1.49 N.A., Nikon), spinning disk confocal unit (CSU-X1, Yokogawa) with super-resolution Live-SR (Gataca) adaptor and sCMOS camera (Prime 95B, Teledyne Photometrics) controlled by NIS-Elements (AR version 5.2, Nikon). For quantitative analysis of MYO15A, WHRN, EPS8, GPSM2, and GNAI3 immunofluorescence, Z-stacks of hair bundles were acquired using identical laser excitation power and detector gain / exposure settings. Images were background subtracted and a 0.5 µm ROI was placed over the individual tips of row 1 stereocilia and the mean intensity calculated per hair bundle. Hair bundle intensities from *+/jd* and *jd/jd* mice were normalized to *+/jd* hair bundles to yield a relative intensity measurement.

### Actin barbed-end incorporation assay

Barbed-end labelling was performed on acutely isolated cochleae, as previously described^43^. Briefly, cochleae from *shaker-2* or *jordan* mice were dissected at P6 in Hank’s Balanced Salt Solution (HBSS, Corning) to remove Reissner’s membrane, stria vascularis and tectorial membrane. Dissected cochleae were then immediately transferred to PCR tubes and gently washed into cytoskeletal extraction buffer at RT (concentrations in mM): HEPES (20), KCl (138), EGTA (3), MgCl_2_ (4), ATP (2), 1% (w/v) bovine serum albumin (BSA), 0.05% (w/v) saponin, pH 7.5. TMR-labelled rabbit muscle actin (Cytoskeleton) was then supplemented to a final concentration of 1 µM and incubated at RT for 5 min. Cochleae were then washed two times into extraction buffer without saponin or ATP, before being fixed for 30 min at RT in 4% paraformaldehyde (EMS Diasum) diluted in PBS. Fixed cochleae were permeabilized in 0.5% (v/v) Triton X-100 in PBS for 30 min at RT, labelled with Alexa Fluor 488 phalloidin (Life Technologies), and mounted using a high-precision #1.5 cover glass (Thorlabs) with Prolong Gold (Life Technologies). Z-stacks were captured using a spinning-disk confocal microscope (described above) and an 100× oil objective (CFI Apochromat TIRF, 1.49 N.A., Nikon). Following background subtraction, orthogonal YZ-projections were generated from z-stacks using NIS-Elements. A 0.5 µm ROI was used to measure the mean intensity of TMR-actin at row 1 and row 2 stereocilia tips, using the phalloidin signal to locate each stereocilia tip. For each hair cell analyzed, the TMR-actin intensity for each stereocilium in rows 1 and 2 was measured and subsequently normalized to the mean TMR-actin signal of rows 1 and 2 combined. Laser power and camera exposure settings were kept identical during image acquisition.

### Expression of EGFP-MYO15A-2 in mammalian cells

The pEGFP-C2-Myo15a-2 and pEGFP-C2-Myo15a-2(sh2) plasmids expressing an N-terminal EGFP fusion with the mouse MYO15A isoform 2 coding sequence (NP_874357.2) were previously reported^24^. The pEGFP-C2-Myo15a-2(jd) plasmid was generated using site directed mutagenesis (QuikChange II, Agilent) to introduce the *jordan* (c.4940 A > G, NM_010862.2:290-10825; p.D1647G, NP_034992.2) non-synonymous substitution. All expression plasmids were verified by Sanger sequencing and prepared as endotoxin-free, transfection grade DNA (NucleoBond Xtra Maxi EF, TakaraBio). HeLa cells (#CCL2) were obtained as an authenticated, low passage stock from the American Type Culture Collection (ATCC). LLC-PK1-CL4 (CL4) cells were a kind gift from Dr. James Bartles at Northwestern University. HeLa and CL4 cells were cultured in high-glucose DMEM (#11995, Life Technologies), supplemented with 1× GlutaMAX (Life Technologies) and 10% FBS (Atlanta Biologicals), and maintained at 37 °C, 5% CO_2_. Transfection of HeLa cells or CL4 cells was performed using Lipofectamine 3000 (Life Technologies) according to the manufacturer’s protocol. pEGFP-C2 (Clontech) was used as an empty vector control. Cells were plated onto fibronectin (10 µg·mL^−1^) coated glass bottom culture dishes (#1.5, MatTek Corp) and allowed to adhere, and in the case of CL4 cells, characteristic island formation could be observed. Cells were fixed in 4% paraformaldehyde and 2% sucrose in PBS for 10 min (EMS Diasum). Fixed cells were permeabilized/blocked in 0.2% (v/v) Triton X-100, 10% (v/v) normal goat serum (NGS) in PBS for 1 h at RT, followed by labelling with rhodamine phalloidin (Life Technologies) and Hoechst 33342 (Life Technologies) in blocking buffer (2% NGS in PBS). Confocal microscopy images were captured as described above.

### Actin purification and labelling

Actin was extracted from rabbit skeletal acetone powder (Pel-Freeze, AZ) in chilled G-buffer (concentrations in mM), Tris-HCl (2), ATP (0.2), CaCl_2_ (0.1), NaN_3_ (1), DTT (1), pH 8, using established protocols^122^. Actin was additionally labelled on Cys 374 using either N-(1-pyrene)-iodoacetamide (Life Technologies), or tetramethylrhodamine-5-maleimide (Adipogen Life Sciences)^58,123^. F-actin used for steady-state ATPase and gliding filaments assays was purified through two rounds of polymerization and depolymerization using ultracentrifugation. F-actin was dialyzed extensively against MOPS (4), MgCl_2_ (1), EGTA (0.1), DTT (1), NaN_3_ (1), pH 7.0, and its concentration measured at 290 nm (ε = 26,600 M^−1^⋅cm^−1^) prior to use. Unlabelled, pyrene- and TMR-labelled G-actin for polymerization studies were further purified by SEC (Superdex 200, Cytiva) using isocratic elution in G-buffer. Fractions were exclusively taken from the falling edge of the chromatogram, to ensure the recovery of monomeric actin. Concentrations and dye-labelling efficiency was determined at 290 nm (actin: ε = 26,600 M^−1^·cm^−1^), 344 nm (pyrene: ε = 22,000 M^−1^·cm^−1^) and 550 nm (rhodamine: ε = 96,900 M^−1^·cm^−1^). Correction factors were applied for pyrene actin, A_corr_ = A_290_–(0.127 * A_344_), and separately for rhodamine actin, A_corr_ = A_290_–(0.208 * A_550_)^58^. Biotinylated skeletal muscle actin (#8109-01, HyperMol, Germany) was rehydrated, dialyzed against G-buffer and cleared by ultracentrifugation for 60 min at 100k × *g* prior to use. For actin polymerization experiments performed without free ATP in solution, G-actin stocks (with 0.2 mM ATP) were desalted (PD SpinTrap G-25, Cytiva) into a modified G-buffer (no ATP): Tris-HCl (2), CaCl_2_ (0.1), NaN_3_ (1), DTT (1), pH 8, immediately prior to use. Desalted G-actin (ATP) monomers were stored on ice and used within 3 h.

### Expression of MYO15A and deafness mutants in *Sf*9 cells using baculovirus

The baculoviral transfer vector pFastbac1 M15-2IQ-EGFP-FLAG, encoding the wild-type mouse MYO15A motor domain (NP_874357.2, aa. 1–743) with two light-chain binding (LCBD) domains as a C-terminal fusion with EGFP and FLAG moieties, was previously described^35^. The expressed protein was 114 kDa. The *jordan* (p.D1647G) and *shaker-2* (p.C1779Y) non-synonymous substitutions (relative to NP_034992.2) were separately introduced into pFastbac1 M15-2IQ-EGFP-FLAG by site-directed mutagenesis (QuikChange II, Agilent) and verified by Sanger sequencing. Plasmid DNA encoding wild-type (M15-wt), *jordan* (M15-jd) and *shaker-2* (M15-sh2) motor was separately transformed into DH10Bac cells (Life Technologies) and recombinant bacmid DNA prepared following the manufacturer’s protocol. First passage (P1) recombinant baculovirus was generated by transfecting *Sf*9 cells (Expression Systems) with bacmid DNA complexed using linear polyethylenimine (PEI MAX, 40,000 MW, Polysciences Inc., PA) at a 12:1 (PEI: DNA) molar ratio. *Sf*9 cells were maintained in suspension culture with ESF-921 medium (Expression Systems) in a shaking incubator at 27 °C. P1 baculovirus was amplified in *Sf*9 cells using low multiplicity of infection (MOI = 0.1) to generate P2 baculovirus for expression. Dual-promoter baculovirus expressing mouse UNC45B + HSP90AA1 chaperones^35^, and bovine smooth muscle essential (MYL6, also referred to as MLC17B / ELC) + chicken regulatory (MYL12B, also referred to as MLC20/RLC) light chains, were previously described^124^. All baculoviruses were titered using an end-point dilution assay and the *Sf*9 Easy Titer cell line^125^. To express M15-wt, *Sf*9 cells were seeded at a density of 2 × 10^6^ cells·mL^−1^ in ESF-921, and infected simultaneously with three baculoviruses at the following MOI: M15-wt (5), UNC45B/HSP90AA1 (5), ELC/RLC (5). The myosin chaperones UNC45B and HSP90AA1 were co-expressed to aid folding, in addition to essential (MYL6) and regulatory (MYL12B) muscle light chains to bind the LCBDs^35,36^. Identical expressions were performed for the M15-jd and M15-sh2 missense variants. Cells were harvested at 48–72 h post-infection by centrifugation at 500 × *g* and flash frozen in liquid nitrogen.

### Purification of the MYO15A motor domain

M15-wt, M15-jd and M15-sh2 motor domains were purified from frozen *Sf*9 cells following established protocols^35,36^. Cell pellets were lysed using a Dounce homogenizer in extraction buffer (concentrations in mM): MOPS (10), NaCl (500), EGTA (1), MgCl_2_ (10), ATP (2), PMSF (0.2), DTT (0.1), NaN_3_ (1), leupeptin (2 μg·mL^−1^), protease inhibitor cocktail (Halt EDTA-free; Thermo Scientific), pH 7.2. Cell lysates were cleared for 30 min at 48k × *g* and the supernatant incubated with FLAG M2 affinity resin (Sigma-Aldrich) for 3 h at 4 °C. FLAG resin was packed into a gravity flow column and washed with a high-salt buffer, MOPS (10), NaCl (500), EGTA (1), MgCl_2_ (5), ATP (1), PMSF (0.1), DTT (0.1), NaN_3_ (1), leupeptin (2 μg·mL^−1^), pH 7.2, followed by a low-salt buffer, MOPS (10), NaCl (60), EGTA (1), PMSF (0.1), DTT (0.1), NaN_3_ (1), leupeptin (2 μg·mL^−1^), pH 7.0. M15-2IQ protein was eluted using low-salt buffer supplemented with 0.2 mg·mL^−1^ 3× FLAG peptide (American Peptide, CA). For preparative scale protein production, FLAG-eluted M15-wt and M15-jd were bound to a strong anion exchanger (5/50 MonoQ GL; Cytiva) using a Purifier 10 chromatography system (GE Healthcare). The column was washed with MOPS (10), NaCl (100), EGTA (1), PMSF (0.1), DTT (1), pH 7.0, and a 160 CV gradient elution performed to 1 M NaCl (100% B). Fractions eluting at ∼31 mS·cm^−1^ were concentrated (10,000 MWCO; Amicon, EMD-Millipore) and further purified using SEC (Superdex 200, Cytiva) with isocratic elution in MOPS (10), KCl (100), EGTA (0.1), NaN_3_ (1), PMSF (0.1), DTT (1), leupeptin (1 μg·mL^−1^), pH 7.0. The M15-2IQ: ELC: RLC complex (1:1:1) eluted as a single peak and was concentrated (10,000 MWCO) before determining complex concentration at 280 nm (*ε* = 88,020 M^−1^·cm^−1^). For assessment of the hydrodynamic radius of purified M15-wt + M15-jd, size standards (HMW + LMW gel filtration calibration kit; Cytiva) were analyzed in parallel using SEC. Due to the heavy aggregation of M15-sh2 following FLAG capture, the IEX step was omitted, and protein was analyzed directly by SEC.

### ATPase assays

Steady-state actin activated ATPase assays were measured using a NADH-coupled assay, as previously reported^35^. Briefly, M15-wt (30 nM) or M15-jd (150 nM) protein was assayed in the following reaction buffer (concentration in mM): MOPS (10), KCl (50), MgCl_2_ (5), EGTA (0.1), MgATP (2), 40 U·mL^−1^ lactate dehydrogenase (Sigma-Aldrich), 200 U·mL^−1^ pyruvate kinase (Sigma-Aldrich), phosphoenolpyruvate(1) (Sigma-Aldrich), NADH (0.2), pH 7.0 at 20 ± 0.1 °C. Due to its lower activity, a higher concentration (150 nM) of M15-2IQ *jordan* protein was used. The concentration of F-actin in the reaction was titrated from 0 to 100 μM. The absorbance of NADH (*ε* = 6220 M^−1^·cm^−1^) at 340 nm was measured using a dual-beam spectrophotometer (UV-1800, Shimadzu) and the ATP hydrolysis rate calculated from the change in absorbance over time. Hydrolysis rates were corrected for basal ATPase activity in the absence of F-actin, and also for the intrinsic ATPase activity of F-actin. ATP hydrolysis rates were fit to the Michaelis-Menten equation to estimate *k*_cat_ and *k*_ATPase_ using Prism (GraphPad v9).

### Gliding filament motility assay

Motility chambers were assembled by coating a clean cover glass (#1.5) with 0.1% nitrocellulose in amyl acetate (Ladd Research Industries), and attaching it to a microscope slide with two strips of double-sided adhesive tape (Scotch, 3 M) to form a channel approximately 3 mm wide. The chamber was incubated for 5 mins in 0.1 mg·mL^−1^ anti-GFP (clone GFP-20, Sigma-Aldrich) diluted with motility buffer (MB) (concentrations in mM): MOPS (20), KCl (10), MgCl_2_ (5), EGTA (0.1), pH 7.0. The surface was blocked using 1 mg·mL^−1^ BSA (Sigma-Aldrich) diluted in MB and incubated for 1 min. After washing the chamber with MB, 1 μM M15-wt or M15-jd diluted in MB was incubated for 1 min to functionalize the anti-GFP coated surface. The chamber was washed with 1 mg·mL^−1^ BSA in MB, followed by MB alone. Finally, the chamber was incubated for 2 mins with TMR-phalloidin stabilized actin filaments (5 nM) diluted in MB, and subsequently washed in MB. Filaments were visualized using an inverted epifluorescence microscope (Olympus IX-51) and motility recorded in MB supplemented with 50 mM DTT, 2 mM ATP, 3 mM glucose, 100 μg⋅mL glucose oxidase, and 20 μg⋅mL catalase at 30 ± 0.5 °C. Actin filament velocities were analyzed as described^52^.

### Pyrene-actin polymerization assay

Actin polymerization was measured using G-actin labelled on Cys 374 with N-(1-pyrene)-iodoacetamide (see above), and a cuvette-based fluorometer (PTI Quantamaster 400, HORIBA Scientific) used to excite pyrene at 365 nm and capture fluorescence emission at 407 nm. Gel-filtered G-actin (10% pyrene labelled, with free ATP, or desalted to yield G-actin(ATP) with no free ATP) was converted to the physiological Mg^2+^ bound form by addition of 50 µM MgCl_2_ and 0.2 mM EGTA for exactly two minutes at room temperature. The polymerization reaction was then initiated by mixing 6 µM G-actin (3× final concentration) with 1.5× KMEI buffer in a 1:2 ratio, respectively. M15-wt or M15-jd was included in the KMEI buffer at 1.5× final concentration as needed. Where applicable, yeast profilin was added to the 3× G-actin stock. Final concentrations were 2 µM G-actin and 0.1–1 µM myosin, 8 µM profilin (optional) in assay buffer (concentrations in mM): KCl (50), MgCl_2_ (1), EGTA (1), imidazole (10), pH 7.0 at 25 ± 0.1 °C. Data were corrected for dead-time and fluorescence recorded until the transient reached plateau, or for a maximum of 3 h. Half times were calculated as described^126^. Initial gradients were calculated using linear regression in the first 200 s of a recording, and expressed relative to the G-actin + KMEI control.

### TIRFM single-filament polymerization assay

High-tolerance coverslips (24 × 50 mm, #1.5, Marienfeld Superior, Germany) were cleaned by sequential sonication (10 min each) in 2% Hellmanex III (Hellma, Germany), 1 M KOH, 100% ethanol, and finally Milli-Q water. Coverslips were dried under a nitrogen stream and processed for 10 min under argon plasma (ZEPTO, Diener Electronic, Germany). A mixture of mPEG-silane (2 mg⋅mL^−1^, Laysan Bio, AL) and biotin–PEG–silane (2 μg ⋅ mL^−1^, Laysan Bio) was prepared in 96% ethanol, 0.1% (v/v) HCl. Coverslips were coated with 100 μL of the mPEG mixture and baked at 70 °C for 1 h. Coverslips were rinsed twice in 96% ethanol, sonicated for 10 min, followed by two rinses in Milli-Q, sonicated for 10 min in Milli-Q, and finally dried under a nitrogen stream. Flow chambers were assembled using double-sided sticky tape to create a 3 mm wide channel on a glass slide. Functionalized coverslips were placed over the tape and firmly pushed down to seal the flow chamber. Immediately prior to use, flow cells were washed with buffer T50 (concentrations in mM): Tris⋅HCl (10), KCl (50), DTT (1), pH 8.0. Flow cells were sequentially washed with 0.1 mg⋅mL^−1^ (NeutrAvidin, Thermo Scientific) in T50 for 1 min, followed by a wash with 1 mg⋅mL^−1^ bovine serum albumin (A0281, Sigma-Aldrich) in T50 for 30 s, and a final wash of T50. Experiments were performed in the following reaction buffer (final concentration in mM): KCl (50), MgCl_2_ (1), EGTA (1), imidazole (10), ATP (0.025), DTT (10), glucose (15), 0.5% methylcellulose, 20 μg⋅mL catalase, 100 μg⋅mL glucose oxidase, pH 7.0. G-actin (1 µM total) was included in the reaction with TMR-actin (20%) and biotin-actin (10%) doping. Purified M15-wt or M15-jd (1 µM) was optionally added, as needed. The reaction was loaded into the flow cell and immediately mounted onto an inverted microscope (Nikon Ti2-E) equipped with an oil immersion objective (CFI Apochromat TIRF 100×, 1.49 N.A., Nikon) for objective-style total internal reflection fluorescence (TIRF) microscopy (H-TIRF, Nikon). The flow cell was illuminated using a 561 nm laser line, and emission filtered using a bandpass filter (ET630/75 m, Chroma). Time-lapse images were captured on an EM-CCD camera (iXon Ultra 888, Andor) controlled by NIS-Elements (AR version 5.2, Nikon). Samples were imaged at 21 ± 1 °C. The assay dead-time was typically 45–60 s, and was included in data analyses.

Images were pre-processed in FIJI (v1.54, https://fiji.sc)^127^ by performing background subtraction and image registration (descriptor-based series registration, 2d/3d + *t*). Actin-filament densities were quantified using the Analyze Particle command (size > 3 pixel^2^, circularity: 0.0–1.0) to count discrete particles within a 50 × 50 μm region of interest (ROI) that were randomly selected from the field of view. A minimum of 3 experiments, from two independent protein preparations, were analyzed for each condition. Filament elongation rates were calculated from time-lapse TIRFM imaging sequences using kymographs generated in NIS-Elements Software (version 5.2, Nikon). At least 40 filaments from two independent experiments were analyzed for each condition.

### Statistical analyses

All statistical calculations and non-linear regressions were performed in Prism (GraphPad). Regression fit parameters are quoted as mean ± SEM, and data points as mean ± SD, unless otherwise stated. All statistical tests were two-tailed and *P*-values are denoted as: * *P* < 0.05, ** *P* < 0.01, *** *P* < 0.001, **** *P* < 0.0001.

### Reporting summary

Further information on research design is available in the Nature Portfolio Reporting Summary linked to this article.

## Supplementary information

Supplementary Information

Description of Additional Supplementary Files

Supplementary Movie 1

Supplementary Movie 2

Reporting Summary

## Source data

Source Data

## Data Availability

The data that support the findings of this study are included in the main text and supplementary information files. Raw data can be obtained from the corresponding authors upon request.  Source data are provided with this paper.

## References

[1] Barr-Gillespie, P.-G. Assembly of hair bundles, an amazing problem for cell biology. *Mol. Biol. Cell* **26**, 2727–2732 (2015). 26229154 10.1091/mbc.E14-04-0940 PMC4571333

[2] Richardson, G. P., de Monvel, J. B. & Petit, C. How the genetics of deafness illuminates auditory physiology. *Annu. Rev. Physiol.* **73**, 311–334 (2011). 21073336 10.1146/annurev-physiol-012110-142228

[3] Vélez-Ortega, A. C. & Frolenkov, G. I. Building and repairing the stereocilia cytoskeleton in mammalian auditory hair cells. *Hear. Res.* **376**, 47–57 (2019). 30638948 10.1016/j.heares.2018.12.012 PMC6456422

[4] Tilney, L. G., Cotanche, D. A. & Tilney, M. S. Actin filaments, stereocilia and hair cells of the bird cochlea. VI. How the number and arrangement of stereocilia are determined. *Dev. Camb. Engl.* **116**, 213–226 (1992). 10.1242/dev.116.1.213 1483389

[5] Kaltenbach, J. A., Falzarano, P. R. & Simpson, T. H. Postnatal development of the hamster cochlea. II. Growth and differentiation of stereocilia bundles. *J. Comp. Neurol.* **350**, 187–198 (1994). 7884037 10.1002/cne.903500204

[6] Krey, J. F. et al. Mechanotransduction-dependent control of stereocilia dimensions and row identity in inner hair cells. *Curr. Biol. CB* **30**, 442–454.e7 (2020). 31902726 10.1016/j.cub.2019.11.076 PMC7002276

[7] Tilney, L. G., Derosier, D. J. & Mulroy, M. J. The organization of actin filaments in the stereocilia of cochlear hair cells. *J. Cell Biol.* **86**, 244–259 (1980). 6893452 10.1083/jcb.86.1.244 PMC2110658

[8] Flock, A. & Cheung, H. C. Actin filaments in sensory hairs of inner ear receptor cells. *J. Cell Biol.* **75**, 339–343 (1977). 318131 10.1083/jcb.75.2.339 PMC2109950

[9] Beurg, M., Fettiplace, R., Nam, J.-H. & Ricci, A. J. Localization of inner hair cell mechanotransducer channels using high-speed calcium imaging. *Nat. Neurosci.* **12**, 553–558 (2009). 19330002 10.1038/nn.2295 PMC2712647

[10] Narayanan, P. et al. Length regulation of mechanosensitive stereocilia depends on very slow actin dynamics and filament-severing proteins. *Nat. Commun.* **6**, 6855 (2015). 25897778 10.1038/ncomms7855 PMC4523390

[11] Zhang, D.-S. et al. Multi-isotope imaging mass spectrometry reveals slow protein turnover in hair-cell stereocilia. *Nature* **481**, 520–524 (2012). 22246323 10.1038/nature10745 PMC3267870

[12] Schneider, M. E., Belyantseva, I. A., Azevedo, R. B. & Kachar, B. Rapid renewal of auditory hair bundles. *Nature* **418**, 837–838 (2002). 12192399 10.1038/418837a

[13] Drummond, M. C. et al. Live-cell imaging of actin dynamics reveals mechanisms of stereocilia length regulation in the inner ear. *Nat. Commun.* **6**, 6873 (2015). 25898120 10.1038/ncomms7873 PMC4411292

[14] Wang, A. et al. Association of unconventional myosin MYO15 mutations with human nonsyndromic deafness DFNB3. *Science* **280**, 1447–1451 (1998). 9603736 10.1126/science.280.5368.1447

[15] Friedman, T. B. et al. A gene for congenital, recessive deafness DFNB3 maps to the pericentromeric region of chromosome 17. *Nat. Genet.* **9**, 86–91 (1995). 7704031 10.1038/ng0195-86

[16] Rehman, A. U. et al. Mutational spectrum of MYO15A and the molecular mechanisms of DFNB3 human deafness. *Hum. Mutat.* **37**, 991–1003 (2016). 27375115 10.1002/humu.23042 PMC5021573

[17] Hartman, M. A. & Spudich, J. A. The myosin superfamily at a glance. *J. Cell Sci.* **125**, 1627–1632 (2012). 22566666 10.1242/jcs.094300 PMC3346823

[18] Houdusse, A. & Sweeney, H. L. How myosin generates force on actin filaments. *Trends Biochem. Sci.* **41**, 989–997 (2016). 27717739 10.1016/j.tibs.2016.09.006 PMC5123969

[19] Moreland, Z. G. & Bird, J. E. Myosin motors in sensory hair bundle assembly. *Curr. Opin. Cell Biol.* **79**, 102132 (2022). 36257241 10.1016/j.ceb.2022.102132 PMC10230610

[20] Houdusse, A. & Titus, M. A. The many roles of myosins in filopodia, microvilli and stereocilia. *Curr. Biol. CB* **31**, R586–R602 (2021). 34033792 10.1016/j.cub.2021.04.005 PMC8149809

[21] Anderson, D. W. et al. The motor and tail regions of myosin XV are critical for normal structure and function of auditory and vestibular hair cells. *Hum. Mol. Genet.* **9**, 1729–1738 (2000). 10915760 10.1093/hmg/9.12.1729

[22] Liang, Y. et al. Characterization of the human and mouse unconventional myosin XV genes responsible for hereditary deafness DFNB3 and shaker 2. *Genomics* **61**, 243–258 (1999). 10552926 10.1006/geno.1999.5976

[23] Fang, Q. et al. The 133-kDa N-terminal domain enables myosin 15 to maintain mechanotransducing stereocilia and is essential for hearing. *eLife* **4**, e08627 (2015). 26302205 10.7554/eLife.08627 PMC4592939

[24] Belyantseva, I. A., Boger, E. T. & Friedman, T. B. Myosin XVa localizes to the tips of inner ear sensory cell stereocilia and is essential for staircase formation of the hair bundle. *Proc. Natl. Acad. Sci. USA.* **100**, 13958–13963 (2003). 14610277 10.1073/pnas.2334417100 PMC283528

[25] Probst, F. J. et al. Correction of deafness in shaker-2 mice by an unconventional myosin in a BAC transgene. *Science* **280**, 1444–1447 (1998). 9603735 10.1126/science.280.5368.1444

[26] Vélez-Ortega, A. C., Freeman, M. J., Indzhykulian, A. A., Grossheim, J. M. & Frolenkov, G. I. Mechanotransduction current is essential for stability of the transducing stereocilia in mammalian auditory hair cells. *eLife* **6**, e24661 (2017). 28350294 10.7554/eLife.24661 PMC5407859

[27] Mburu, P. et al. Defects in whirlin, a PDZ domain molecule involved in stereocilia elongation, cause deafness in the whirler mouse and families with DFNB31. *Nat. Genet.* **34**, 421–428 (2003). 12833159 10.1038/ng1208

[28] Belyantseva, I. A. et al. Myosin-XVa is required for tip localization of whirlin and differential elongation of hair-cell stereocilia. *Nat. Cell Biol.* **7**, 148–156 (2005). 15654330 10.1038/ncb1219

[29] Delprat, B. et al. Myosin XVa and whirlin, two deafness gene products required for hair bundle growth, are located at the stereocilia tips and interact directly. *Hum. Mol. Genet.* **14**, 401–410 (2005). 15590698 10.1093/hmg/ddi036

[30] Manor, U. et al. Regulation of stereocilia length by myosin XVa and whirlin depends on the actin-regulatory protein Eps8. *Curr. Biol. CB* **21**, 167–172 (2011). 21236676 10.1016/j.cub.2010.12.046 PMC3040242

[31] Zampini, V. et al. Eps8 regulates hair bundle length and functional maturation of mammalian auditory hair cells. *PLoS Biol* **9**, e1001048 (2011). 21526224 10.1371/journal.pbio.1001048 PMC3079587

[32] Mauriac, S. A. et al. Defective Gpsm2/Gαi3 signalling disrupts stereocilia development and growth cone actin dynamics in Chudley-McCullough syndrome. *Nat. Commun.* **8**, 14907 (2017). 28387217 10.1038/ncomms14907 PMC5385604

[33] Tarchini, B., Tadenev, A. L. D., Devanney, N. & Cayouette, M. A link between planar polarity and staircase-like bundle architecture in hair cells. *Dev. Camb. Engl.* **143**, 3926–3932 (2016). 10.1242/dev.139089 PMC6514398 27660326

[34] Tadenev, A. L. D. et al. GPSM2-GNAI specifies the tallest stereocilia and defines hair bundle row identity. *Curr. Biol. CB* **29**, 921–934.e4 (2019). 30827920 10.1016/j.cub.2019.01.051 PMC6516530

[35] Bird, J. E. et al. Chaperone-enhanced purification of unconventional myosin 15, a molecular motor specialized for stereocilia protein trafficking. *Proc. Natl. Acad. Sci. USA.* **111**, 12390–12395 (2014). 25114250 10.1073/pnas.1409459111 PMC4151768

[36] Jiang, F. et al. The ATPase mechanism of myosin 15, the molecular motor mutated in DFNB3 human deafness. *J. Biol. Chem.* **296**, 100243 (2021). 33372036 10.1074/jbc.RA120.014903 PMC7948958

[37] Gong, R. et al. Structural basis for tunable control of actin dynamics by myosin-15 in mechanosensory stereocilia. *Sci. Adv.* **8**, eabl4733 (2022). 10.1126/sciadv.abl4733 PMC9299544 35857845

[38] Potter, P. K. et al. Novel gene function revealed by mouse mutagenesis screens for models of age-related disease. *Nat. Commun.* **7**, 12444 (2016). 27534441 10.1038/ncomms12444 PMC4992138

[39] Stepanyan, R., Belyantseva, I. A., Griffith, A. J., Friedman, T. B. & Frolenkov, G. I. Auditory mechanotransduction in the absence of functional myosin-XVa. *J. Physiol.* **576**, 801–808 (2006). 16973713 10.1113/jphysiol.2006.118547 PMC1890419

[40] Rzadzinska, A. K., Schneider, M. E., Davies, C., Riordan, G. P. & Kachar, B. An actin molecular treadmill and myosins maintain stereocilia functional architecture and self-renewal. *J. Cell Biol.* **164**, 887–897 (2004). 15024034 10.1083/jcb.200310055 PMC2172292

[41] Peng, A. W., Belyantseva, I. A., Hsu, P. D., Friedman, T. B. & Heller, S. Twinfilin 2 regulates actin filament lengths in cochlear stereocilia. *J. Neurosci. Off. J. Soc. Neurosci.* **29**, 15083–15088 (2009). 10.1523/JNEUROSCI.2782-09.2009 PMC2823077 19955359

[42] Avenarius, M. R. et al. Heterodimeric capping protein is required for stereocilia length and width regulation. *J. Cell Biol.* **216**, 3861–3881 (2017). 28899994 10.1083/jcb.201704171 PMC5674897

[43] McGrath, J. et al. Actin at stereocilia tips is regulated by mechanotransduction and ADF/cofilin. *Curr. Biol. CB* **31**, 1141–1153.e7 (2021). 33400922 10.1016/j.cub.2020.12.006 PMC8793668

[44] Krey, J. F. et al. Control of stereocilia length during development of hair bundles. *PLoS Biol.* **21**, e3001964 (2023). 37011103 10.1371/journal.pbio.3001964 PMC10101650

[45] Kerber, M. L. et al. A novel form of motility in filopodia revealed by imaging myosin-X at the single-molecule level. *Curr. Biol. CB* **19**, 967–973 (2009). 19398338 10.1016/j.cub.2009.03.067 PMC2817954

[46] Bird, J. E. et al. Harnessing molecular motors for nanoscale pulldown in live cells. *Mol. Biol. Cell* **28**, 463–475 (2017). 27932498 10.1091/mbc.E16-08-0583 PMC5341729

[47] Brawley, C. M. & Rock, R. S. Unconventional myosin traffic in cells reveals a selective actin cytoskeleton. *Proc. Natl. Acad. Sci. USA.* **106**, 9685–9690 (2009). 19478066 10.1073/pnas.0810451106 PMC2701044

[48] Nagy, S. et al. A myosin motor that selects bundled actin for motility. *Proc. Natl. Acad. Sci. USA.* **105**, 9616–9620 (2008). 18599451 10.1073/pnas.0802592105 PMC2474510

[49] Reymann, A.-C. et al. Actin network architecture can determine myosin motor activity. *Science* **336**, 1310–1314 (2012). 22679097 10.1126/science.1221708 PMC3649007

[50] Ropars, V. et al. The myosin X motor is optimized for movement on actin bundles. *Nat. Commun.* **7**, 12456 (2016). 27580874 10.1038/ncomms12456 PMC5025751

[51] Zheng, L., Zheng, J., Whitlon, D. S., García-Añoveros, J. & Bartles, J. R. Targeting of the hair cell proteins cadherin 23, harmonin, myosin XVa, espin, and prestin in an epithelial cell model. *J. Neurosci. Off. J. Soc. Neurosci.* **30**, 7187–7201 (2010). 10.1523/JNEUROSCI.0852-10.2010 PMC2989820 20505086

[52] Sellers, J. R., Cuda, G., Wang, F. & Homsher, E. Myosin-specific adaptations of the motility assay. *Methods Cell Biol.* **39**, 23–49 (1993). 8246800 10.1016/s0091-679x(08)60159-4

[53] Fievez, S. & Carlier, M. F. Conformational changes in subdomain-2 of G-actin upon polymerization into F-actin and upon binding myosin subfragment-1. *FEBS Lett.* **316**, 186–190 (1993). 8420804 10.1016/0014-5793(93)81212-i

[54] Lheureux, K., Forné, T. & Chaussepied, P. Interaction and polymerization of the G-actin-myosin head complex: effect of DNase I. *Biochemistry* **32**, 10005–10014 (1993). 8399127 10.1021/bi00089a016

[55] Miller, L., Phillips, M. & Reisler, E. Polymerization of G-actin by myosin subfragment 1. *J. Biol. Chem.* **263**, 1996–2002 (1988). 2962999

[56] Yagi, K., Mase, R., Sakakibara, I. & Asai, H. Function of heavy meromyosin in the acceleration of actin polymerization. *J. Biol. Chem.* **240**, 2448–2454 (1965). 14304851

[57] Cooper, J. A., Walker, S. B. & Pollard, T. D. Pyrene actin: documentation of the validity of a sensitive assay for actin polymerization. *J. Muscle Res. Cell Motil.* **4**, 253–262 (1983). 6863518 10.1007/BF00712034

[58] Fujiwara, I., Takahashi, S., Tadakuma, H., Funatsu, T. & Ishiwata, S. Microscopic analysis of polymerization dynamics with individual actin filaments. *Nat. Cell Biol.* **4**, 666–673 (2002). 12198494 10.1038/ncb841

[59] Kuhn, J. R. & Pollard, T. D. Real-time measurements of actin filament polymerization by total internal reflection fluorescence microscopy. *Biophys. J.* **88**, 1387–1402 (2005). 15556992 10.1529/biophysj.104.047399 PMC1305141

[60] Fievez, S., Carlier, M. F. & Pantaloni, D. Mechanism of myosin subfragment-1-induced assembly of CaG-actin and MgG-actin into F-actin-S1-decorated filaments. *Biochemistry* **36**, 11843–11850 (1997). 9305976 10.1021/bi971206o

[61] Fievez, S., Pantaloni, D. & Carlier, M. F. Kinetics of myosin subfragment-1-induced condensation of G-actin into oligomers, precursors in the assembly of F-actin-S1. Role of the tightly bound metal ion and ATP hydrolysis. *Biochemistry* **36**, 11837–11842 (1997). 9305975 10.1021/bi971205w

[62] Dominguez, R. & Holmes, K. C. Actin structure and function. *Annu. Rev. Biophys.* **40**, 169–186 (2011). 21314430 10.1146/annurev-biophys-042910-155359 PMC3130349

[63] Avraham, K. B. et al. The mouse Snell’s waltzer deafness gene encodes an unconventional myosin required for structural integrity of inner ear hair cells. *Nat. Genet.* **11**, 369–375 (1995). 7493015 10.1038/ng1295-369

[64] Gibson, F. et al. A type VII myosin encoded by the mouse deafness gene shaker-1. *Nature* **374**, 62–64 (1995). 7870172 10.1038/374062a0

[65] Gillespie, P. G. & Corey, D. P. Myosin and adaptation by hair cells. *Neuron* **19**, 955–958 (1997). 9390509 10.1016/s0896-6273(00)80387-6

[66] Hasson, T. et al. Unconventional myosins in inner-ear sensory epithelia. *J. Cell Biol.* **137**, 1287–1307 (1997). 9182663 10.1083/jcb.137.6.1287 PMC2132524

[67] Holt, J. R. et al. A chemical-genetic strategy implicates myosin-1c in adaptation by hair cells. *Cell* **108**, 371–381 (2002). 11853671 10.1016/s0092-8674(02)00629-3

[68] Salles, F. T. et al. Myosin IIIa boosts elongation of stereocilia by transporting espin 1 to the plus ends of actin filaments. *Nat. Cell Biol.* **11**, 443–450 (2009). 19287378 10.1038/ncb1851 PMC2750890

[69] Ebrahim, S. et al. Stereocilia-staircase spacing is influenced by myosin III motors and their cargos espin-1 and espin-like. *Nat. Commun.* **7**, 10833 (2016). 26926603 10.1038/ncomms10833 PMC4773517

[70] Geli, M. I., Lombardi, R., Schmelzl, B. & Riezman, H. An intact SH3 domain is required for myosin I-induced actin polymerization. *EMBO J.* **19**, 4281–4291 (2000). 10944111 10.1093/emboj/19.16.4281 PMC302045

[71] Sun, Y., Martin, A. C. & Drubin, D. G. Endocytic internalization in budding yeast requires coordinated actin nucleation and myosin motor activity. *Dev. Cell* **11**, 33–46 (2006). 16824951 10.1016/j.devcel.2006.05.008

[72] Fitz, G. N., Weck, M. L., Bodnya, C., Perkins, O. L. & Tyska, M. J. Protrusion growth driven by myosin-generated force. *Dev. Cell* **58**, 18–33.e6 (2023). 36626869 10.1016/j.devcel.2022.12.001 PMC9940483

[73] Cirilo, J. A., Liao, X., Perrin, B. J. & Yengo, C. M. The dynamics of actin protrusions can be controlled by tip-localized myosin motors. *J. Biol. Chem.* **300**, 105516 (2024). 38042485 10.1016/j.jbc.2023.105516 PMC10801316

[74] Gunther, L. K., Cirilo, J. A., Desetty, R. & Yengo, C. M. Deafness mutation in the MYO3A motor domain impairs actin protrusion elongation mechanism. *Mol. Biol. Cell* **33**, ar5 (2022). 34788109 10.1091/mbc.E21-05-0232 PMC8886822

[75] Quinlan, M. E., Heuser, J. E., Kerkhoff, E. & Mullins, R. D. Drosophila spire is an actin nucleation factor. *Nature* **433**, 382–388 (2005). 15674283 10.1038/nature03241

[76] Mullins, R. D., Stafford, W. F. & Pollard, T. D. Structure, subunit topology, and actin-binding activity of the Arp2/3 complex from Acanthamoeba. *J. Cell Biol.* **136**, 331–343 (1997). 9015304 10.1083/jcb.136.2.331 PMC2134809

[77] Mullins, R. D., Heuser, J. A. & Pollard, T. D. The interaction of Arp2/3 complex with actin: nucleation, high affinity pointed end capping, and formation of branching networks of filaments. *Proc. Natl. Acad. Sci. USA.* **95**, 6181–6186 (1998). 9600938 10.1073/pnas.95.11.6181 PMC27619

[78] Machesky, L. M., Atkinson, S. J., Ampe, C., Vandekerckhove, J. & Pollard, T. D. Purification of a cortical complex containing two unconventional actins from Acanthamoeba by affinity chromatography on profilin-agarose. *J. Cell Biol.* **127**, 107–115 (1994). 7929556 10.1083/jcb.127.1.107 PMC2120189

[79] Sagot, I., Rodal, A. A., Moseley, J., Goode, B. L. & Pellman, D. An actin nucleation mechanism mediated by Bni1 and profilin. *Nat. Cell Biol.* **4**, 626–631 (2002). 12134165 10.1038/ncb834

[80] Ahuja, R. et al. Cordon-bleu is an actin nucleation factor and controls neuronal morphology. *Cell* **131**, 337–350 (2007). 17956734 10.1016/j.cell.2007.08.030 PMC2507594

[81] Chereau, D. et al. Leiomodin is an actin filament nucleator in muscle cells. *Science* **320**, 239–243 (2008). 18403713 10.1126/science.1155313 PMC2845909

[82] Shin, J.-B. et al. Hair bundles are specialized for ATP delivery via creatine kinase. *Neuron* **53**, 371–386 (2007). 17270734 10.1016/j.neuron.2006.12.021 PMC1839076

[83] Krey, J. F. & Barr-Gillespie, P. G. Molecular composition of vestibular hair bundles. *Cold Spring Harb. Perspect. Med.* **9**, a033209 (2019). 29844221 10.1101/cshperspect.a033209 PMC6314077

[84] Dumont, R. A. et al. Plasma membrane Ca2+-ATPase isoform 2a is the PMCA of hair bundles. *J. Neurosci. Off. J. Soc. Neurosci.* **21**, 5066–5078 (2001). 10.1523/JNEUROSCI.21-14-05066.2001 PMC6762840 11438582

[85] Reynolds, M. J., Hachicho, C., Carl, A. G., Gong, R. & Alushin, G. M. Bending forces and nucleotide state jointly regulate F-actin structure. *Nature* **611**, 380–386 (2022). 36289330 10.1038/s41586-022-05366-w PMC9646526

[86] Li, F. & Higgs, H. N. The mouse Formin mDia1 is a potent actin nucleation factor regulated by autoinhibition. *Curr. Biol. CB* **13**, 1335–1340 (2003). 12906795 10.1016/s0960-9822(03)00540-2

[87] Lin, L. et al. Phase separation-mediated condensation of Whirlin-Myo15-Eps8 stereocilia tip complex. *Cell Rep.* **34**, 108770 (2021). 33626355 10.1016/j.celrep.2021.108770

[88] Shi, Y., Lin, L., Wang, C. & Zhu, J. Promotion of row 1-specific tip complex condensates by Gpsm2-Gαi provides insights into row identity of the tallest stereocilia. *Sci. Adv.* **8**, eabn4556 (2022). 35687681 10.1126/sciadv.abn4556 PMC9187228

[89] Effertz, T., Becker, L., Peng, A. W. & Ricci, A. J. Phosphoinositol-4,5-bisphosphate regulates auditory hair-cell mechanotransduction-channel pore properties and fast adaptation. *J. Neurosci. Off. J. Soc. Neurosci.* **37**, 11632–11646 (2017). 10.1523/JNEUROSCI.1351-17.2017 PMC5707765 29066559

[90] Goñi, G. M. et al. Phosphatidylinositol 4,5-bisphosphate triggers activation of focal adhesion kinase by inducing clustering and conformational changes. *Proc. Natl. Acad. Sci. USA* **111**, E3177–E3186 (2014). 25049397 10.1073/pnas.1317022111 PMC4128148

[91] Neidt, E. M., Scott, B. J. & Kovar, D. R. Formin differentially utilizes profilin isoforms to rapidly assemble actin filaments. *J. Biol. Chem.* **284**, 673–684 (2009). 18978356 10.1074/jbc.M804201200

[92] Shin, J.-B. et al. Molecular architecture of the chick vestibular hair bundle. *Nat. Neurosci.* **16**, 365–374 (2013). 23334578 10.1038/nn.3312 PMC3581746

[93] Zhu, Y. et al. Single-cell proteomics reveals changes in expression during hair-cell development. *eLife* **8**, e50777 (2019). 31682227 10.7554/eLife.50777 PMC6855842

[94] Clayton, J. E., Pollard, L. W., Murray, G. G. & Lord, M. Myosin motor isoforms direct specification of actomyosin function by tropomyosins. *Cytoskeleton* **72**, 131–145 (2015). 25712463 10.1002/cm.21213 PMC4414888

[95] Ricca, B. L. & Rock, R. S. The stepping pattern of myosin X is adapted for processive motility on bundled actin. *Biophys. J.* **99**, 1818–1826 (2010). 20858426 10.1016/j.bpj.2010.06.066 PMC2941030

[96] Santos, A., Shauchuk, Y., Cichoń, U., Vavra, K. C. & Rock, R. S. How actin tracks affect myosin motors. *Adv. Exp. Med. Biol.* **1239**, 183–197 (2020). 32451860 10.1007/978-3-030-38062-5_9

[97] Loomis, P. A. et al. Espin cross-links cause the elongation of microvillus-type parallel actin bundles in vivo. *J. Cell Biol.* **163**, 1045–1055 (2003). 14657236 10.1083/jcb.200309093 PMC2173610

[98] Mallavarapu, A. & Mitchison, T. Regulated actin cytoskeleton assembly at filopodium tips controls their extension and retraction. *J. Cell Biol.* **146**, 1097–1106 (1999). 10477762 10.1083/jcb.146.5.1097 PMC2169471

[99] Murphy, D. B., Gray, R. O., Grasser, W. A. & Pollard, T. D. Direct demonstration of actin filament annealing in vitro. *J. Cell Biol.* **106**, 1947–1954 (1988). 3384850 10.1083/jcb.106.6.1947 PMC2115120

[100] Kinosian, H. J., Selden, L. A., Estes, J. E. & Gershman, L. C. Actin filament annealing in the presence of ATP and phalloidin. *Biochemistry* **32**, 12353–12357 (1993). 8241122 10.1021/bi00097a011

[101] Sept, D., Xu, J., Pollard, T. D. & McCammon, J. A. Annealing accounts for the length of actin filaments formed by spontaneous polymerization. *Biophys. J.* **77**, 2911–2919 (1999). 10585915 10.1016/s0006-3495(99)77124-9 PMC1300564

[102] Young, G. et al. Quantitative mass imaging of single biological macromolecules. *Science* **360**, 423–427 (2018). 29700264 10.1126/science.aar5839 PMC6103225

[103] Hundt, N. et al. Direct observation of the molecular mechanism underlying protein polymerization. *Sci. Adv.* **8**, eabm7935 (2022). 36044567 10.1126/sciadv.abm7935 PMC9432825

[104] Okreglak, V. & Drubin, D. G. Loss of Aip1 reveals a role in maintaining the actin monomer pool and an in vivo oligomer assembly pathway. *J. Cell Biol.* **188**, 769–777 (2010). 20231387 10.1083/jcb.200909176 PMC2845081

[105] Heissler, S. M. & Sellers, J. R. Various themes of myosin regulation. *J. Mol. Biol.* **428**, 1927–1946 (2016). 26827725 10.1016/j.jmb.2016.01.022 PMC4860093

[106] Thirumurugan, K., Sakamoto, T., Hammer, J. A., Sellers, J. R. & Knight, P. J. The cargo-binding domain regulates structure and activity of myosin 5. *Nature* **442**, 212–215 (2006). 16838021 10.1038/nature04865 PMC1852638

[107] Liu, J., Taylor, D. W., Krementsova, E. B., Trybus, K. M. & Taylor, K. A. Three-dimensional structure of the myosin V inhibited state by cryoelectron tomography. *Nature* **442**, 208–211 (2006). 16625208 10.1038/nature04719

[108] Sato, O., Li, X.-D. & Ikebe, M. Myosin Va becomes a low duty ratio motor in the inhibited form. *J. Biol. Chem.* **282**, 13228–13239 (2007). 17363376 10.1074/jbc.M610766200

[109] Sckolnick, M., Krementsova, E. B., Warshaw, D. M. & Trybus, K. M. More than just a cargo adapter, melanophilin prolongs and slows processive runs of myosin Va. *J. Biol. Chem.* **288**, 29313–29322 (2013). 23979131 10.1074/jbc.M113.476929 PMC3795233

[110] Li, X.-D., Ikebe, R. & Ikebe, M. Activation of myosin Va function by melanophilin, a specific docking partner of myosin Va. *J. Biol. Chem.* **280**, 17815–17822 (2005). 15760894 10.1074/jbc.M413295200

[111] Umeki, N. et al. The tail binds to the head-neck domain, inhibiting ATPase activity of myosin VIIA. *Proc. Natl. Acad. Sci. USA.* **106**, 8483–8488 (2009). 19423668 10.1073/pnas.0812930106 PMC2688991

[112] Yang, Y. et al. A FERM domain autoregulates Drosophila myosin 7a activity. *Proc. Natl. Acad. Sci. USA.* **106**, 4189–4194 (2009). 19255446 10.1073/pnas.0808682106 PMC2649957

[113] Umeki, N. et al. Phospholipid-dependent regulation of the motor activity of myosin X. *Nat. Struct. Mol. Biol.* **18**, 783–788 (2011). 21666676 10.1038/nsmb.2065

[114] Sakai, T., Umeki, N., Ikebe, R. & Ikebe, M. Cargo binding activates myosin VIIA motor function in cells. *Proc. Natl. Acad. Sci. USA.* **108**, 7028–7033 (2011). 21482763 10.1073/pnas.1009188108 PMC3084129

[115] Holló, A. et al. Molecular regulatory mechanism of human myosin-7a. *J. Biol. Chem.* **299**, 105243 (2023). 37690683 10.1016/j.jbc.2023.105243 PMC10579538

[116] Hardisty-Hughes, R. E., Parker, A. & Brown, S. D. M. A hearing and vestibular phenotyping pipeline to identify mouse mutants with hearing impairment. *Nat. Protoc.* **5**, 177–190 (2010). 20057387 10.1038/nprot.2009.204

[117] Mianné, J. et al. Correction of the auditory phenotype in C57BL/6N mice via CRISPR/Cas9-mediated homology directed repair. *Genome Med.* **8**, 16 (2016). 26876963 10.1186/s13073-016-0273-4 PMC4753642

[118] Dunbar, L. A. et al. Clarin-2 is essential for hearing by maintaining stereocilia integrity and function. *EMBO Mol. Med.* **11**, e10288 (2019). 31448880 10.15252/emmm.201910288 PMC6728604

[119] Katsuno, T. et al. TRIOBP-5 sculpts stereocilia rootlets and stiffens supporting cells enabling hearing. *JCI Insight* **4**, e128561 (2019). 128561. 31217345 10.1172/jci.insight.128561 PMC6629139

[120] Bariani, P., De Chiffre, L., Hansen, H. N. & Horsewell, A. Investigation on the traceability of three dimensional scanning electron microscope measurements based on the stereo-pair technique. *Precis. Eng.* **29**, 219–228 (2005).

[121] Ezan, J. et al. Primary cilium migration depends on G-protein signalling control of subapical cytoskeleton. *Nat. Cell Biol.* **15**, 1107–1115 (2013). 23934215 10.1038/ncb2819

[122] Spudich, J. A. & Watt, S. The regulation of rabbit skeletal muscle contraction. I. Biochemical studies of the interaction of the tropomyosin-troponin complex with actin and the proteolytic fragments of myosin. *J. Biol. Chem.* **246**, 4866–4871 (1971). 4254541

[123] Criddle, A. H., Geeves, M. A. & Jeffries, T. The use of actin labelled with N-(1-pyrenyl)iodoacetamide to study the interaction of actin with myosin subfragments and troponin/tropomyosin. *Biochem. J.* **232**, 343–349 (1985). 3911945 10.1042/bj2320343 PMC1152885

[124] Pato, M. D., Sellers, J. R., Preston, Y. A., Harvey, E. V. & Adelstein, R. S. Baculovirus expression of chicken nonmuscle heavy meromyosin II-B. Characterization of alternatively spliced isoforms. *J. Biol. Chem.* **271**, 2689–2695 (1996). 8576242 10.1074/jbc.271.5.2689

[125] Hopkins, R. & Esposito, D. A rapid method for titrating baculovirus stocks using the Sf-9 Easy Titer cell line. *BioTechniques* **47**, 785–788 (2009). 19852765 10.2144/000113238

[126] Hansen, S. D., Zuchero, J. B. & Mullins, R. D. Cytoplasmic actin: purification and single molecule assembly assays. *Methods Mol. Biol.* **1046**, 145–170 (2013). 23868587 10.1007/978-1-62703-538-5_9 PMC4013826

[127] Schindelin, J. et al. Fiji: an open-source platform for biological-image analysis. *Nat. Methods* **9**, 676–682 (2012). 22743772 10.1038/nmeth.2019 PMC3855844

