# Supplementary material for: Myosin-based nucleation of actin filaments contributes to stereocilia development critical for hearing
Source: Nat Commun. 2025 Jan 22;16:947. doi: 10.1038/s41467-025-55898-8 (PMC11754657; doi:10.1038/s41467-025-55898-8)
Supplement: Supplementary file 1 — Supplementary Information [file 41467_2025_55898_MOESM1_ESM.pdf]

SUPPLEMENTARY INFORMATION

Myosin-based Nucleation of Actin Filaments Contributes to Stereocilia Development Critical for Hearing

Zane G. Moreland, Fangfang Jiang, Carlos Aguilar, Melanie Barzik, Rui Gong, Ghazaleh Behnammanesh, Jinho Park, Arik Shams, Christian Faaborg-Andersen, Jesse C. Werth, Randall Harley, Daniel C. Sutton, James B. Heidings, Stacey M. Cole, Andrew Parker, Susan Morse, Elizabeth Wilson, Yasuharu Takagi, James R. Sellers, Steve D.M. Brown, Thomas B. Friedman, Gregory M. Alushin, Michael R. Bowl & Jonathan E. Bird

Contents:

- Supplementary Figure 1
- Supplementary Figure 2
- Supplementary Figure 3

Supplementary Figure 1

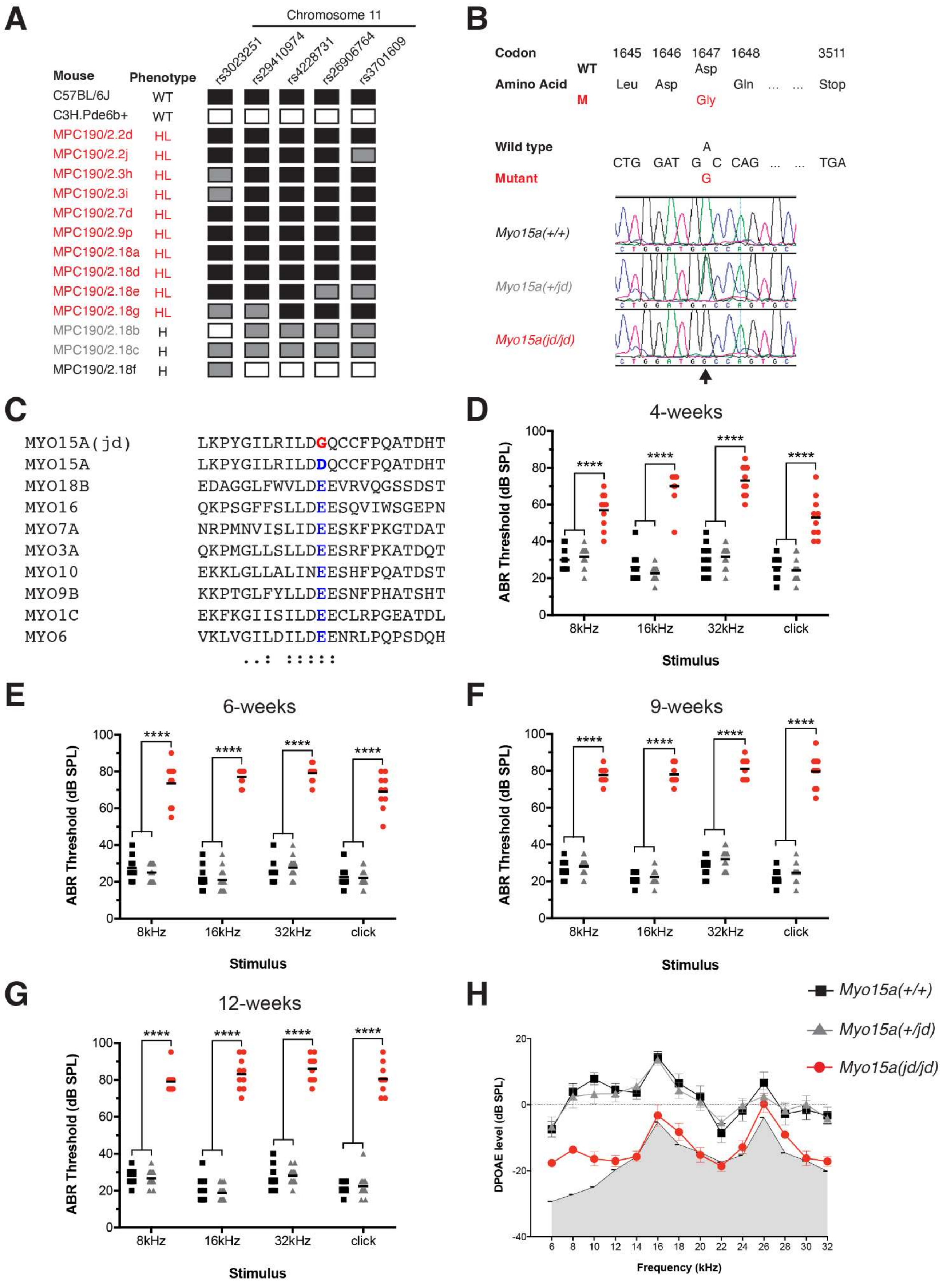

Supplementary Figure 1. Mapping of the *Myo15a jordan* mutation and detailed ABR phenotyping data.

A) Whole genome SNP mapping of genomic DNA from ten MPC190 mice exhibiting hearing loss (red, HL) and three hearing (H) littermate controls. The genotype of each mouse is either homozygous for C57BL/6J (black box), C3H (white box) or heterozygous (grey box) for each marker. The analysis defined a 16.5Mb critical interval on chromosome 11 between markers rs29410974 and rs26906764 (Chr11:50420012-67162951, GRCm38). **B)** Sanger sequencing confirmed the only WGS-identified, coding lesion (arrow) within the critical interval, in exon 17 (ENSMUSE00000244718) of the *Myo15a* gene (ENSMUSG00000042678). A nucleotide transition (c.4940A>G) at codon 1647 alters the wild-type aspartate (D) codon to a mutant glycine (G). Example electropherograms are shown for *Myo15a*$^{+/+}$, *Myo15a*$^{+/jd}$ and *Myo15a*$^{jd/jd}$. **C)** ClustalW alignment of the mutated *jordan* residue in MYO15A with other mouse unconventional myosin proteins. **D-G)** Minimum ABR detection thresholds for a longitudinal cohort of *Myo15a*$^{jd/jd}$ mice (n = 10 mice), *Myo15a*$^{+/+}$ (n=10 mice) and *Myo15a*$^{+/jd}$ (n=15 mice) littermates at 4, 6, 9 and 12 weeks reveal progressive hearing loss. **** P < 0.0001; two-way ANOVA with Tukey's multiple comparisons test. **H)** Distortion Product Otoacoustic Emission (DPOAE) responses are greatly reduced at 12 weeks in *Myo15a*$^{jd/jd}$ mutants (red, n = 10 mice) at all frequencies tested compared with *Myo15a*$^{+/+}$ (black, n = 9 mice) and *Myo15a*$^{+/jd}$ (grey, n = 14 mice) littermates. Data are mean ± SD.

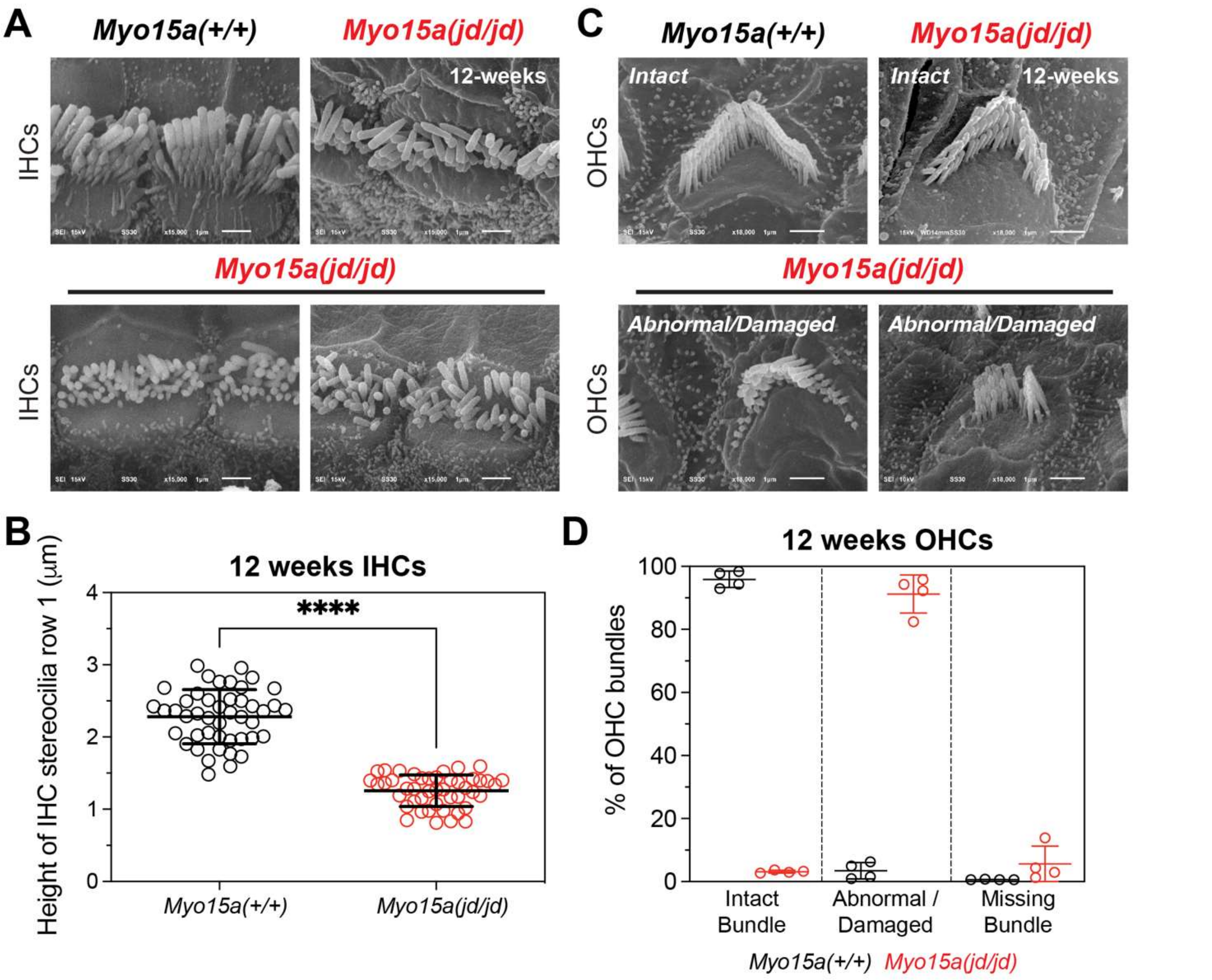

Supplementary Figure 2. Hair bundle architecture is disrupted at 12 weeks in *jordan* hair cells. **A)** SEM analyses of IHC bundles from *Myo15a*$^{jd/jd}$ and *Myo15a*$^{+/+}$ littermates at 12 weeks. **B)** Quantification of the tallest (row 1) stereocilia at 12 weeks in IHCs from *Myo15a*$^{+/+}$ mice (45 stereocilia from 3 animals) and *Myo15a*$^{jd/jd}$ mice (45 stereocilia from 3 animals). **** $P < 0.0001$, unpaired, two-tailed *t*-test. **C)** SEM analyses of *Myo15a*$^{jd/jd}$ OHC bundles at 12 weeks. OHC bundles were qualitatively categorized as "intact", "abnormal/damaged" or "missing". Representative examples are shown. In the "abnormal/damaged" group, stereocilia were typically resorbed from the periphery of the hair bundle. **D)** Quantification of OHC bundle categories at 12 weeks in *Myo15a*$^{+/+}$ (1344 OHCs from 4 animals) and *Myo15a*$^{jd/jd}$ littermates (972 OHCs from 4 animals). Data are mean ± SD. All images and quantification are from the mid-cochlear turn. Scale bars, 1 μm.

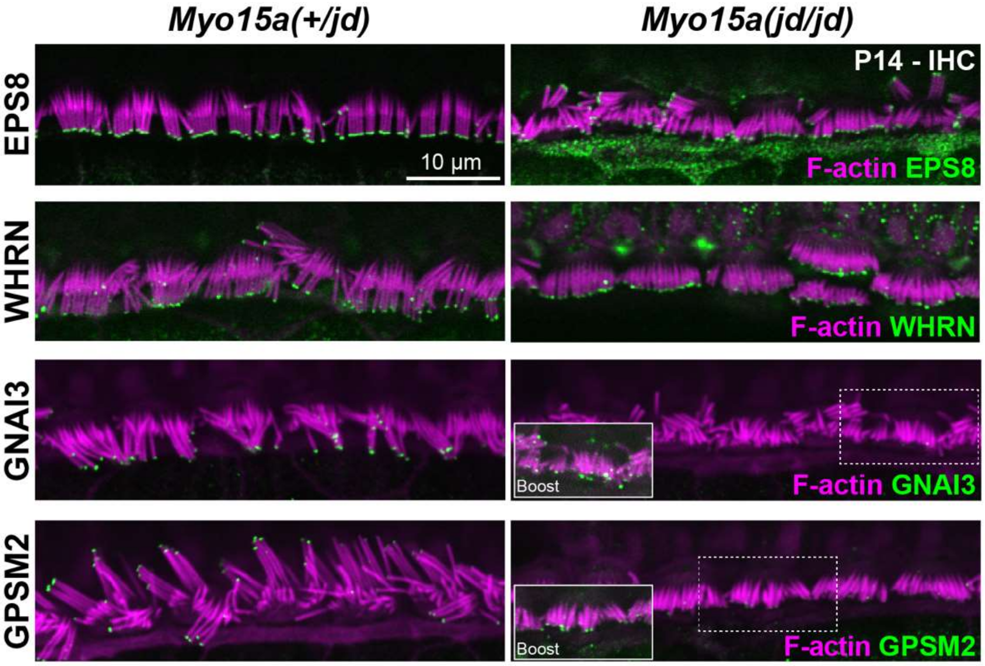

Supplementary Figure 3. Targeting of elongation complex proteins is altered in *jordan* hair cells at P14. Immunofluorescence (IF) confocal images of anti-EPS8, anti-WHRN, anti-GNAI3 labelling or anti-GPSM2 (green) in control *Myo15a*$^{+/jd}$ and *Myo15a*$^{jd/jd}$ IHCs fixed at P14. Phalloidin was used to label F-actin (magenta). Matched antibody images for *+/jd* and *jd/jd* genotypes have been mapped equally, except for inset panels where the image contrast has been boosted. Images are representative of data from two independent animals per genotype and antibody combination. Scale bars, 10 µm.
