# Supplementary material for: Myosin-based nucleation of actin filaments contributes to stereocilia development critical for hearing
Source: Nat Commun. 2025 Jan 22;16:947. doi: 10.1038/s41467-025-55898-8 (PMC11754657; doi:10.1038/s41467-025-55898-8)
Supplement: Supplementary file 2 — Description of Additional Supplementary Files [file 41467_2025_55898_MOESM2_ESM.pdf]

Description of Additional Supplementary Files

File name: Supplementary Movie 1
Description: Time-lapse TIRF microscopy of MYO15A-induced actin polymerization in the presence of ATP. Total internal reflection fluorescence (TIRF) microscopy of 1 µM G-actin (20% rhodamine, 10% biotin labelled) polymerizing on a PEG-biotin-NeutrAvidin functionalized cover glass. KMEI buffer (top panel), KMEI + 1 µM M15-wt (middle panel), or KMEI + M15-jd (bottom panel) were added at 0 seconds. Time stamps (in seconds) are relative to the assay initiation at t = 0 s and are corrected for the dead time. Rhodamine fluorescence is shown with an inverted lookup table. Scale bars, 10 µm.

File name: Supplementary Movie 2
Description: Time-lapse TIRF microscopy of MYO15A-induced nucleation in the absence of ATP. Total internal reflection fluorescence (TIRF) microscopy of 1 µM G-actin (20% rhodamine, 10% biotin labelled) polymerizing on a PEG-biotin-NeutrAvidin functionalized cover glass. KMEI buffer (top panel), KMEI + 1 µM M15-wt (middle panel), or KMEI + 1 µM M15-jd (bottom panel) were added at 0 seconds. Time stamps (in seconds) are relative to the assay initiation at t = 0 s and are corrected for the dead time. Rhodamine fluorescence is shown with an inverted lookup table. Scale bars, 10 µm.
