# Supplementary material for: Myosin-based nucleation of actin filaments contributes to stereocilia development critical for hearing
Source: Nat Commun. 2025 Jan 22;16:947. doi: 10.1038/s41467-025-55898-8 (PMC11754657; doi:10.1038/s41467-025-55898-8)
Supplement: Supplementary file 5 — Reporting Summary [file 41467_2025_55898_MOESM5_ESM.pdf]

nature portfolio

Corresponding author(s): Jonathan Bird & Michael Bowl

Last updated by author(s): 12/2/2024

Reporting Summary

Nature Portfolio wishes to improve the reproducibility of the work that we publish. This form provides structure for consistency and transparency in reporting. For further information on Nature Portfolio policies, see our Editorial Policies and the Editorial Policy Checklist.

Statistics

For all statistical analyses, confirm that the following items are present in the figure legend, table legend, main text, or Methods section.

n/a	Confirmed	
☐	☒	The exact sample size (*n*) for each experimental group/condition, given as a discrete number and unit of measurement
☐	☒	A statement on whether measurements were taken from distinct samples or whether the same sample was measured repeatedly
☐	☒	The statistical test(s) used AND whether they are one- or two-sided<br>*Only common tests should be described solely by name; describe more complex techniques in the Methods section.*
☒	☐	A description of all covariates tested
☐	☒	A description of any assumptions or corrections, such as tests of normality and adjustment for multiple comparisons
☐	☒	A full description of the statistical parameters including central tendency (e.g. means) or other basic estimates (e.g. regression coefficient) AND variation (e.g. standard deviation) or associated estimates of uncertainty (e.g. confidence intervals)
☐	☒	For null hypothesis testing, the test statistic (e.g. *F*, *t*, *r*) with confidence intervals, effect sizes, degrees of freedom and *P* value noted<br>*Give P values as exact values whenever suitable.*
☒	☐	For Bayesian analysis, information on the choice of priors and Markov chain Monte Carlo settings
☒	☐	For hierarchical and complex designs, identification of the appropriate level for tests and full reporting of outcomes
☒	☐	Estimates of effect sizes (e.g. Cohen's *d*, Pearson's *r*), indicating how they were calculated

Our web collection on statistics for biologists contains articles on many of the points above.

Software and code

Policy information about availability of computer code

Data collection	Auditory brainstem responses were collected, amplified and averaged using TDT System 3 in conjunction with either BioSig RP (version 4.4.11) or BioSig RZ (version 5.7.1) software. Distortion product oto-acoustic emissions tests were performed with the TDT RZ6 System 3 hardware and BioSig RZ (version 5.7.1) software. Microscopy image capture was performed using Zeiss software (Zen Blue), or Nikon software (NIS-Elements AR). FPLC liquid chromatography data was collected using the AKTA UNICORN software suite. Spectroscopic measurements of ATPase rates were collected using the Shimadzu UVProbe software. Fluorometry data were collected using the Photon Technology International FelixGX software. No custom code was used.
Data analysis	Image data were analysed using open-source ImageJ and FIJI software packages, in additional to Nikon NIS-Elements AR. Numerical data were tabulated and processed using Microsoft Excel and statistical testing performed using GraphPad Prism. No custom code was used.

For manuscripts utilizing custom algorithms or software that are central to the research but not yet described in published literature, software must be made available to editors and reviewers. We strongly encourage code deposition in a community repository (e.g. GitHub). See the Nature Portfolio guidelines for submitting code & software for further information.

Data

Policy information about availability of data

All manuscripts must include a data availability statement. This statement should provide the following information, where applicable:

- Accession codes, unique identifiers, or web links for publicly available datasets
- A description of any restrictions on data availability
- For clinical datasets or third party data, please ensure that the statement adheres to our policy

The data that support the findings of this study are included in the main text and supplementary information files. Raw data can be obtained from the corresponding authors upon request. Source data are provided with this manuscript.

Research involving human participants, their data, or biological material

Policy information about studies with human participants or human data. See also policy information about sex, gender (identity/presentation), and sexual orientation and race, ethnicity and racism.

Reporting on sex and gender	*Use the terms sex (biological attribute) and gender (shaped by social and cultural circumstances) carefully in order to avoid confusing both terms. Indicate if findings apply to only one sex or gender; describe whether sex and gender were considered in study design; whether sex and/or gender was determined based on self-reporting or assigned and methods used. Provide in the source data disaggregated sex and gender data, where this information has been collected, and if consent has been obtained for sharing of individual-level data; provide overall numbers in this Reporting Summary. Please state if this information has not been collected. Report sex- and gender-based analyses where performed, justify reasons for lack of sex- and gender-based analysis.*
Reporting on race, ethnicity, or other socially relevant groupings	*Please specify the socially constructed or socially relevant categorization variable(s) used in your manuscript and explain why they were used. Please note that such variables should not be used as proxies for other socially constructed/relevant variables (for example, race or ethnicity should not be used as a proxy for socioeconomic status). Provide clear definitions of the relevant terms used, how they were provided (by the participants/respondents, the researchers, or third parties), and the method(s) used to classify people into the different categories (e.g. self-report, census or administrative data, social media data, etc.) Please provide details about how you controlled for confounding variables in your analyses.*
Population characteristics	*Describe the covariate-relevant population characteristics of the human research participants (e.g. age, genotypic information, past and current diagnosis and treatment categories). If you filled out the behavioural & social sciences study design questions and have nothing to add here, write "See above."*
Recruitment	*Describe how participants were recruited. Outline any potential self-selection bias or other biases that may be present and how these are likely to impact results.*
Ethics oversight	*Identify the organization(s) that approved the study protocol.*

Note that full information on the approval of the study protocol must also be provided in the manuscript.

Field-specific reporting

Please select the one below that is the best fit for your research. If you are not sure, read the appropriate sections before making your selection.

☒ Life sciences ☐ Behavioural & social sciences ☐ Ecological, evolutionary & environmental sciences

For a reference copy of the document with all sections, see nature.com/documents/nr-reporting-summary-flat.pdf

Life sciences study design

All studies must disclose on these points even when the disclosure is negative.

Sample size	The jordan mouse pedigree was identified from the phenotype-driven ENU-mutagenesis Harwell Aging Screen, wherein large cohorts of mice (n > 50-100) were screened for phenotypes of interest (including hearing loss). Utilizing the data obtained from the jordan cohort, we used GraphPad StatMate to carry out power calculations to determine sample size in an unpaired t-test using the standard deviation of the measured hearing thresholds, a significance level of p=0.01 (two-tailed), and a power of 95%. As such, the effect size was estimated using real data corresponding to the jordan hearing loss phenotype compared with the hearing thresholds of wild-type and heterozygote littermates. This calculation determined that a cohort size of ≥ 7 mice/genotype would be sufficient to detect a 30 dB SPL threshold difference in hearing sensitivity.<br><br>No predetermination of sample sizes by power analysis was performed for stereocilia length measurements (Figure 2), hair cell immunofluoresence (Figure 3), cell transfection (Figure 4), ATPase steady-state or velocity measurements (Figure 5), or actin polymerization experiments (Figures 6 + 7). For these experiments, we did not have a priori information of the expected effect, or effect sizes, and instead used sample sizes established in our prior work, and the work of others in the field, to establish the reproducibility of the measurement. We believe that the sample sizes in the manuscript are sufficient as the variation measured between experimental conditions is small compared the overall effect sizes reported.

Data exclusions	No data were excluded from analyses.
Replication	All analyses reported were replicated in at least biological duplicates for immunofluorescence (with similar results), and in at least triplicate for all other experiments (with similar results). Replication is reported as "independent determinations" in all figures.
Randomization	The jordan mutant mice and littermates were allocated into groups based on genotype.
Blinding	For ABR and DPOAE hearing tests, mice were housed in cages containing mixtures of genotypes, and mice from these cages were tested in a randomized order. At the point of data collection the operator was blinded to genotype. Moreover, the recorded primary data for jordan ABRs (i.e. the ABR waveform traces) were viewed blinded and re-scored by a second operator.<br><br>We could not blind experimenters to mouse genotype during hair cell imaging / analysis, since the phenotypes of the hair bundles were distinctive enough that the experiment was effectively unblinded. Protein experiments were not possible to blind as the effect sizes between wild-type and mutant Myo15 were large.

Reporting for specific materials, systems and methods

We require information from authors about some types of materials, experimental systems and methods used in many studies. Here, indicate whether each material, system or method listed is relevant to your study. If you are not sure if a list item applies to your research, read the appropriate section before selecting a response.

Materials & experimental systems

n/a	Involved in the study
☐	☒ Antibodies
☐	☒ Eukaryotic cell lines
☒	☐ Palaeontology and archaeology
☐	☒ Animals and other organisms
☒	☐ Clinical data
☒	☐ Dual use research of concern
☒	☐ Plants

Methods

n/a	Involved in the study
☒	☐ ChIP-seq
☒	☐ Flow cytometry
☒	☐ MRI-based neuroimaging

Antibodies

Antibodies used	PB48 rabbit anti-MYO15A (custom produced antibody available from Jonathan Bird, UF). HL5136 rabbit anti-WHRN (custom produced antibody available from Thomas B. Friedman, NIDCD/NIH). EPS8: mouse anti-EPS8 (#610143, BD Biosciences). GPSM2: rabbit anti-GPSM2 (custom produced antibody available from Mireille Montcouquiol, INSERM, France). GNAI3: rabbit anti-GNAI3 (#G4040, Sigma-Aldrich).
Validation	Specificity of all antibodies for immunofluorescence was validated in previous publications; see Belyantseva 2003 (PMID 14610277); Belyantseva 2005 (PMID 15654330); Manor 2011 (PMID 21236676); Mauriac 2017 (PMID 28387217).

Eukaryotic cell lines

Policy information about cell lines and Sex and Gender in Research

Cell line source(s)	HeLa (obtained from the ATCC: CCL-2); LLC-PK1-CL4 (RRID:CVCL_IP75, obtained from Dr. James Bartles, Northwestern University). Sf9 (RRID: CVCL_0549, obtained from Expression Systems, CA).
Authentication	HeLa cells were directly obtained from the ATCC as an authenticated cell line and kept as a low passage stock. The LLC-PK1-CL4 has a specific cellular phenotype (production of microvilli) that was visually confirmed, but this stock was not validated by STR markers. Sf9 insect cells were obtained as authenticated low passage stocks directly from Expression Systems.
Mycoplasma contamination	All cell lines tested negative for mycoplasma contamination.
Commonly misidentified lines<br>(See ICLAC register)	None used in this study.

Animals and other research organisms

Policy information about studies involving animals; ARRIVE guidelines recommended for reporting animal research, and Sex and Gender in Research

Laboratory animals	The Myo15 jordan mutant mouse was originally identified from the MRC Harwell Institute phenotype-driven N-ethyl-N-nitrosourea (ENU) Ageing Screen (Potter et al., 2016). The original G3 mice (MPC190) were on a mixed C57BL/6J and C3H.Pde6b+ genetic background, and mice of both sexes were equally affected regards their hearing deficit. At MRC Harwell, further investigation of the jordan auditory phenotype was undertaken on >G9 mice backcrossed to C57BL/6N, with auditory phenotyping by ABR performed at

	4-, 6-, 9- and 12-weeks of age, and by DPOAE at 12-weeks of age. Concurrently, Myo15 jordan mice were imported to the NIH and the University of Florida (UF) and maintained on a 'sightless C3H' (C3H.Pde6brd1) background. Myo15 shaker 2 were obtained from the Jackson Laboratory (Stock No: 000109) and maintained on a C57BL/6J background.
Wild animals	The study did not include wild animals.
Reporting on sex	Both sexes were included in auditory mouse testing and were equally affected regarding their hearing loss. As a result, we did not explicitly consider sex as a biological variable in other mouse experiments using immunofluorescence of scanning electron microscopy.
Field-collected samples	The study did not include samples collected from the field.
Ethics oversight	At MRC Harwell, all animal studies were licensed by the Home Office under the Animals (Scientific Procedures) Act 1986, United Kingdom, and additionally approved by the MRC Harwell Animal Welfare and Ethical Review Body (AWERB). In the US, animal procedures were approved by the Institutional Animal Care and Use Committees (IACUC) at UF (#201910739 to JEB) and at the NIH (#1263-15 to TBF).

Note that full information on the approval of the study protocol must also be provided in the manuscript.

Plants

Seed stocks	*Report on the source of all seed stocks or other plant material used. If applicable, state the seed stock centre and catalogue number. If plant specimens were collected from the field, describe the collection location, date and sampling procedures.*
Novel plant genotypes	*Describe the methods by which all novel plant genotypes were produced. This includes those generated by transgenic approaches, gene editing, chemical/radiation-based mutagenesis and hybridization. For transgenic lines, describe the transformation method, the number of independent lines analyzed and the generation upon which experiments were performed. For gene-edited lines, describe the editor used, the endogenous sequence targeted for editing, the targeting guide RNA sequence (if applicable) and how the editor was applied.*
Authentication	*Describe any authentication procedures for each seed stock used or novel genotype generated. Describe any experiments used to assess the effect of a mutation and, where applicable, how potential secondary effects (e.g. second site T-DNA insertions, mosiacism, off-target gene editing) were examined.*
